# Synthesis of unsymmetrically substituted triarylamines *via* acceptorless dehydrogenative aromatization using a Pd/C and *p*-toluenesulfonic acid hybrid relay catalyst†

**DOI:** 10.1039/c9sc06442g

**Published:** 2020-03-25

**Authors:** Satoshi Takayama, Takafumi Yatabe, Yu Koizumi, Xiongjie Jin, Kyoko Nozaki, Noritaka Mizuno, Kazuya Yamaguchi

**Affiliations:** Department of Applied Chemistry, School of Engineering, The University of Tokyo 7-3-1 Hongo, Bunkyo-ku Tokyo 113-8656 Japan kyama@appchem.t.u-tokyo.ac.jp +81-3-5841-7220; Department of Chemistry and Biotechnology, School of Engineering, The University of Tokyo 7-3-1 Hongo, Bunkyo-ku Tokyo 113-8656 Japan

## Abstract

An efficient and convenient procedure for synthesizing triarylamines based on a dehydrogenative aromatization strategy has been developed. A hybrid relay catalyst comprising carbon-supported Pd (Pd/C) and *p*-toluenesulfonic acid (TsOH) was found to be effective for synthesizing a variety of triarylamines bearing different aryl groups starting from arylamines (diarylamines or anilines), using cyclohexanones as the arylation sources under acceptorless conditions with the release of gaseous H_2_. The proposed reaction comprises the following relay steps: condensation of arylamines and cyclohexanones to produce imines or enamines, dehydrogenative aromatization of the imines or enamines over Pd nanoparticles (NPs), and elimination of H_2_ from the Pd NPs. In this study, an interesting finding was obtained indicating that TsOH may promote the dehydrogenation.

## Abstract 2

An efficient and convenient procedure for synthesizing triarylamines based on a dehydrogenative aromatization strategy has been developed.

## Graphical abstract

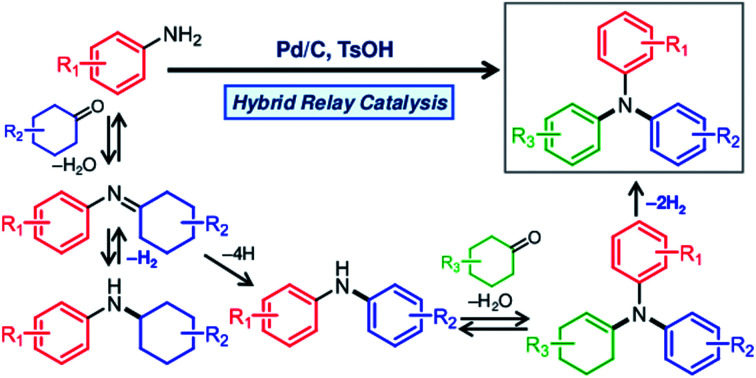

## Introduction

Triarylamines are an important class of chemicals that are widely used for synthesizing valuable materials including polymers, pharmaceuticals, dyes, and natural products.^1^ In addition, they have been utilized as hole transport materials for organic electroluminescent devices and dye-sensitized solar cells.^2^ In general, triarylamines that are effective as hole transport materials often possess three different aryl groups.^2^ Such triarylamines have classically been synthesized by a two-step process in which diarylamines are synthesized first and then coupled with iodobenzenes by the Ullmann reaction.^3^ In recent years, triarylamine synthesis by the Buchwald–Hartwig coupling in the presence of homogeneous Pd catalysts with intricately designed ligands has become mainstream.^4^ Although various triarylamines can be produced by these synthetic methods, the use of haloarenes and the formation of metal halide byproducts are inevitable. Therefore, it is very important to develop new greener methods that can be used to synthesize triarylamines from various starting materials.

**Fig. 1:**
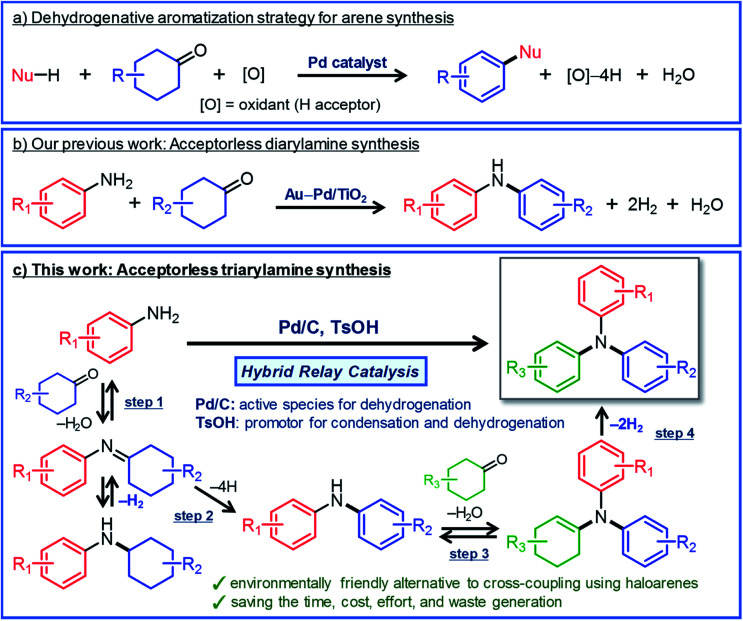
(a) Dehydrogenative aromatization strategy for synthesizing various arenes from six-membered carbocyclic compounds, (b) our recent work on the synthesis of diarylamines from anilines and cyclohexanones *via* acceptorless dehydrogenative aromatization,^12*a*^ and (c) the synthesis of unsymmetrically substituted triarylamines *via* acceptorless dehydrogenative aromatization utilizing hybrid relay catalysis.

Here, we describe a new strategy for synthesizing unsymmetrically substituted triarylamines based on dehydrogenative aromatization. Dehydrogenative aromatization has recently emerged as an attractive method for synthesizing various arenes from ubiquitous saturated six-membered carbocyclic compounds.^5^ Over the past few years, various kinds of synthetically useful catalytic reactions employing dehydrogenative aromatization have been developed, which can be used to effectively access phenols,^6^ aryl ethers,^7^*N*-substituted anilines,^8^ biaryls,^9^ and heterocycles^10^ (Fig. 1a). Recently, Li and co-workers have developed a dearomatization–rearomatization strategy in which phenol substrates are once dearomatized followed by reacting with nucleophiles, and then rearomatized to produce various arene products.^11^ This dearomatization–rearomatization strategy has a great potential to be able to utilize biomass-derived phenols for organic synthesis, and is expected to expand in the future.

For dehydrogenative aromatization reactions, a stoichiometric amount of a hydrogen acceptor is often required. In contrast, acceptorless dehydrogenative aromatization, which generates gaseous H_2_ as a co-product, represents a more environmentally friendly and economical method for synthesizing arenes. Thus far, we have developed several acceptorless dehydrogenative aromatization reactions using supported Pd-based nanoparticle (NP) catalysts;^12^ for example, in the presence of supported Au–Pd alloy NP catalysts (for example, Au–Pd/TiO_2_), unsymmetrically substituted diarylamines with a variety of substituent patterns were synthesized starting from anilines and cyclohexanones (Fig. 1b).^12*a*^ This method was expanded to other combinations of substrates such as cyclohexylamines/cyclohexanones and nitrobenzenes/cyclohexanols.^12*a*^ However, in the Au–Pd alloy NP-catalyzed system, diarylamines were obtained as the major products, and triarylamines were only produced in very low yield (<1%) (Fig. 1b).^12*a*^

If efficient acceptorless arylation of diarylamines using cyclohexanones can be achieved, then environmentally friendly one-pot sequential synthesis of triarylamines with various substitution patterns and co-production of H_2_ should be possible by using a variety of substrate combinations. However, to date, an efficient acceptorless dehydrogenative aromatization system to produce triarylamines from diarylamines and cyclohexanones has not been realized.^13,14^ There are several critical issues facing selective triarylamine production. Because of the low nucleophilicity of diarylamines, condensation with cyclohexanones (step 3 in Fig. 1) is very inefficient. Furthermore, acceptorless dehydrogenation of the corresponding enamines (step 4 in Fig. 1) is unknown. In fact, when the reaction between 3-methyldiphenylamine (**1a**) and 4-ethylcyclohexanone (**2a**) was carried out using a commercially available carbon-supported Pd (Pd/C) catalyst, the yield of the desired unsymmetrically substituted triarylamine **3a** was quite low (see Table 1, entry 1, and related discussion). To realize the effective triarylamine production, acceleration of the condensation and/or the successive dehydrogenation is very important. We hypothesized that suitable acid co-catalysts promote the reaction. To examine the feasibility of our hypothesis, we investigated the effect of acid co-catalysts thoroughly. As a result, we found that various triarylamines could be synthesized from an equimolar mixture of diarylamines and cyclohexanones using a hybrid relay catalyst comprising Pd/C and *p*-toluenesulfonic acid (TsOH) (Fig. 1c). Furthermore, one-pot sequential syntheses of unsymmetrically substituted triarylamines from anilines and two different cyclohexanones were also successful (Fig. 1c). In the proposed one-pot triarylamine synthesis, the absence of isolation/purification of the diarylamine intermediates reduces time, cost, effort, and waste generation. Therefore, the present system has the potential to be an environmentally friendly alternative to cross-coupling reactions using haloarenes. We revealed that triarylamines are produced *via* direct dehydrogenation of enamine intermediates, whereas diarylamines from anilines and cyclohexanones are formed *via* disproportionation of imine intermediates. It is worth noting that an interesting finding was obtained indicating that TsOH may promote the dehydrogenation.

**Table 1:** Screening of catalysts and co-catalysts^a^

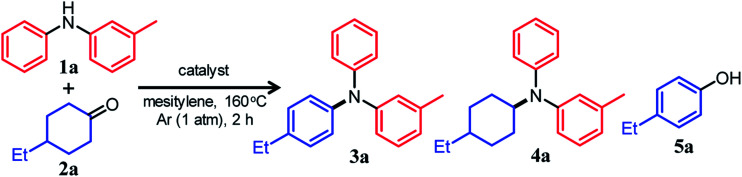					
Entry	Catalyst	Co-catalysts	Yield (%)		
			**3a**	**4a**	**5a**
1	Pd/C	w/o	6	<1	4
**2**	**Pd/C**	**TsOH**	**85**	**<1**	**<1**
3	Pd/C	PhCOOH	7	<1	5
4	Pd/C	CF_3_COOH	13	<1	4
5	Pd/C	CH_3_SO_3_H	20	<1	<1
6	Pd/C	Ti(O^i^Pr)_4_	8	<1	3
7	Pd/C	Al(O^i^Pr)_3_	5	<1	6
8	Pd/C	ZnCl_2_	<1	<1	<1
9	Pd/C	B(C_6_F_5_)_3_	6	<1	<1
10	Pd/C	Sc(O_3_SCF_3_)_3_	1	<1	1
11	Pd/C	Na_2_CO_3_	2	<1	7
12	Pd/C	K_2_CO_3_	1	<1	69
13	Pd/TiO_2_	TsOH	54	<1	<1
14	Pd/Al_2_O_3_	TsOH	4	<1	<1
15	Pd/CeO_2_	TsOH	11	<1	1
16	Pd/LDH	TsOH	<1	<1	9
17	Ru/C	TsOH	3	<1	<1
18	Rh/C	TsOH	54	13	<1
19	Pt/C	TsOH	39	31	<1

a Reaction conditions: catalyst (metal: 2 mol%), additive (10 mol%), **1a** (0.5 mmol), **2a** (0.5 mmol), mesitylene (2 mL), Ar (1 atm), temperature 160 °C, and time 2 h. Yields were determined by GC and are relative to **1a**.

## Results and discussion

### Reaction between 3-methyldiphenylamine and 4-ethylcyclohexanone

We conducted the reaction between 3-methyldiphenylamine (**1a**) and 4-ethylcyclohexanone (**2a**) under various conditions. Initially, the reaction between **1a** and **2a** in mesitylene at 160 °C was carried out in the presence of Pd/C without any co-catalysts. However, the yield of and selectivity to the desired unsymmetrically substituted triarylamine **3a** were unsatisfactory (Table 1, entry 1). We concluded that the condensation between **1a** and **2a** did not proceed well, and in order to solve the problem, we performed the reaction in the presence of various acid or base co-catalysts. As a result, TsOH was identified as a suitable acid co-catalyst. In the presence of Pd/C (2 mol%) and TsOH (10 mol%), the reaction gave the desired triarylamine **3a** in 85% yield after 2 h, and side products such as the reductive amination product **4a** and 4-ethylphenol (**5a**) were hardly detected (Table 1, entry 2). It should be noted that the reaction proceeded efficiently with an equimolar mixture of **1a** and **2a**. It was confirmed by volumetric measurement and mass spectrometry (MS) analysis of the evolved gas that approximately two equivalents of H_2_ gas with respect to **3a** were produced during the reaction. Other Brønsted acid co-catalysts, such as benzoic acid (PhCOOH), trifluoroacetic acid (CF_3_COOH), and methanesulfonic acid (CH_3_SO_3_H), were less effective (Table 1, entries 3–5). The relatively high solubility of TsOH in mesitylene compared with the other Brønsted acid co-catalysts tested is considered to be one reason for the effectiveness of TsOH. For the reaction between **1a** and **2a**, Lewis acid co-catalysts were ineffective (Table 1, entries 6–10). Conversely, the direct dehydrogenation of **2a** was conspicuously promoted in the presence of base co-catalysts (especially K_2_CO_3_), resulting in the undesirable formation of phenol **5a** as the major product (Table 1, entries 11 and 12).^15^

The choice of support was very significant, and the effect of TsOH was most noticeable when using Pd/C (Table 1, entry 2). We prepared several supported Pd catalysts using various oxide or hydroxide supports (Pd/support; support = TiO_2_, Al_2_O_3_, CeO_2_, or Mg_6_Al_2_(OH)_16_CO_3_·4H_2_O (LDH)) and performed the reaction between **1a** and **2a** using these catalysts. Although the Pd content and average particle sizes of the Pd NPs in the Pd/support catalysts were almost the same as those in Pd/C (Fig. S1, Table S1, ESI†), the catalytic performance of all other Pd/support catalysts was greatly inferior to that of Pd/C (Table 1, entries 13–16). Thus, it is presumed that the promotion effect of TsOH cannot be fully exhibited when using Pd/support because of its adsorption on the basic sites of the oxide or hydroxide surface. In particular, when a strong basic support (LDH) was used, the desired **3a** was not produced at all, and the corresponding phenol **5a** was obtained as the major product (Table 1, entry 16).^15^ Therefore, the use of basic supports is inappropriate for this reaction. Among the solvents examined, mesitylene yielded the best results (Table S2, entry 1, ESI†), and decane and diethyleneglycol dimethyl ether (diglyme) also afforded **3a** in good yields (Table S2, entries 2 and 3, ESI†). On the other hand, highly polar solvents such as *N*,*N*-dimethylacetamide and *N*-methylpyrrolidone were not effective (Table S2, entries 4 and 5, ESI†). When the reaction temperature was examined in the range 130–160 °C, it was found that the reaction progressed more efficiently as the temperature increased (Table S3, ESI†).

We also examined the reaction between **1a** and **2a** using supported metal catalysts other than Pd. In the presence of Ru/C, the reaction hardly proceeded (Table 1, entry 17). Conducting the reaction in the presence of Rh/C or Pt/C afforded **3a** in moderate yields along with significant amounts of the reductive amination product **4a** (Table 1, entries 18 and 19). Therefore, Pd/C was determined to be the best catalyst for this reaction. Hereafter, we mainly used the most effective Pd/C and TsOH hybrid relay catalyst under the aforementioned optimized conditions for further detailed investigations.

To establish whether the observed catalysis of the synthesis of **3a** from **1a** and **2a** occurred heterogeneously on Pd/C or was a result of the presence of leached Pd species in the reaction solution, the Pd/C catalyst was removed by hot filtration 1 h after the reaction was started, and the reaction was restarted with the filtrate under the same conditions. As shown in Fig. S2, ESI,† the production of **3a** immediately ceased upon the removal of Pd/C. Additionally, after the reaction, the filtrate was analyzed using inductively coupled plasma atomic emission spectroscopy (ICP-AES), and it was found that Pd species were barely detectable (less than 0.09% of Pd used for the reaction). Consequently, it was concluded that the observed catalysis of the reaction was truly heterogeneous.^16^ Furthermore, the Pd/C retrieved after the reaction could be reused for the reaction between **1a** and **2a**, though the catalytic performance slightly declined; a yield of 72% for **3a** was obtained with a reused Pd/C catalyst (Fig. S3, ESI†).

### Triarylamine synthesis from diarylamines and cyclohexanones

**Fig. 2:**
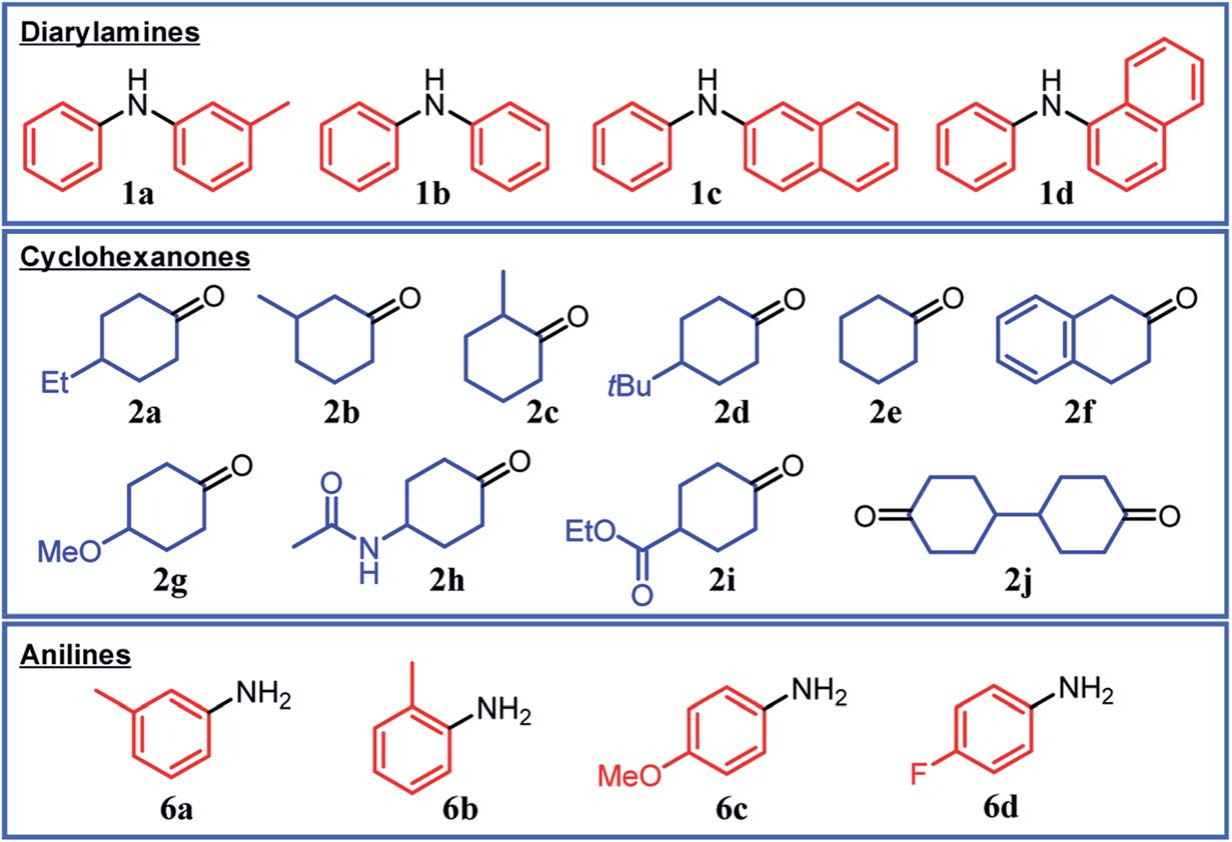
Substrates used in this study.

**Fig. 3:**
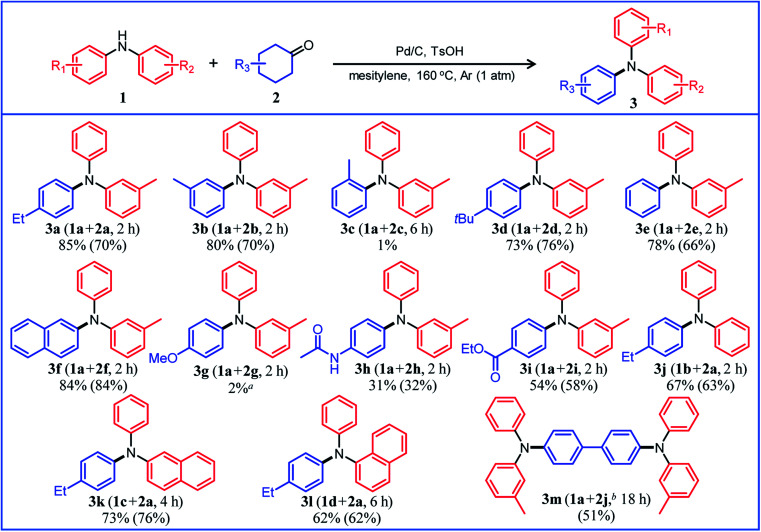
Substrate scope of the proposed triarylamine synthesis from diarylamines and cyclohexanones using a Pd/C and TsOH hybrid relay catalyst. Reaction conditions: Pd/C (2 mol%), TsOH (10 mol%), **1** (0.5 mmol), **2** (0.5 mmol), mesitylene (2 mL), Ar (1 atm), and temperature 160 °C. Yields are relative to **1**. GC yields and isolated yields (values in parentheses) are shown. In all cases, phenol side products **5** were hardly detected. ^*a*^Elimination of the methoxy group occurred during the reaction, giving **3e** as the major product (35%). ^*b*^**1a** (1.0 mmol), **2j** (0.5 mmol).

Under the optimized reaction conditions described above using Pd/C and TsOH, we next examined the scope of suitable substrates for the proposed triarylamine synthesis from diarylamines and cyclohexanones. The substrates used in this study are shown in Fig. 2. As summarized in Fig. 3, various kinds of substrate combinations were converted into the corresponding unsymmetrically substituted triarylamines in moderate-to-high yields. These reactions (except for the synthesis of **3c** and **3g**) efficiently proceeded using equimolar mixtures of diarylamines and cyclohexanones. The desired triarylamine products were readily isolated by simple column chromatography on silica gel.

Cyclohexanone (**2e**) and its derivatives with alkyl groups at the 3- or 4-position (**2a**, **2b**, and **2d**) smoothly reacted with diarylamine **1a**, affording the corresponding triarylamine derivatives in high yields (**3a**, **3b**, **3d**, and **3e**). Unfortunately, the use of 2-methylcyclohexanone (**2c**) as the arylation source was difficult; for the reaction between **1a** and **2c**, the desired triarylamine **3c** was hardly produced. We speculated that if there is a substituent at the 2-position, the steric hindrance of the cyclohexene ring to be dehydrogenated will increase, thus impeding the access of the enamine intermediate to the Pd NP surface. However, if a substituent is introduced at the 2-position of aniline in advance, it is possible to synthesize triarylamines bearing a substituent at the 2-position such as **3c**, as discussed in more detail later.

By using tetrahydronaphthalenone (**2f**), it was possible to introduce a naphthalene skeleton into the diarylamine (**3f**). When using 4-methoxycyclohexanone (**2g**) as the arylation source, an undesirable elimination of the methoxy group occurred during the aromatization step, giving **3e** as the major product. In order to synthesize triarylamines bearing a methoxy group such as **3g**, it is indispensable to introduce the methoxy group into the aniline in advance, as discussed in more detail later. Cyclohexanones bearing an amide (**2h**) or an ester group (**2i**) were tested with the proposed system, affording the corresponding triarylamines in moderate yields (**3h** and **3i**). Diphenylamine (**1b**) and naphthylphenylamines (**1c** and **1d**) also worked well as reaction partners of **2a**, affording the corresponding triarylamines in good yields (**3j**, **3k**, and **3l**). It is noteworthy that TPD (**3m**), which is a highly valuable hole transport material for organic electroluminescent devices,^2^ was readily synthesized from **1a** and bis(4-cyclohexanone) **2j** (**1a** : **2j** = 2 : 1) utilizing the proposed method.

### One-pot triarylamine synthesis from anilines and two different cyclohexanones

**Fig. 4:**
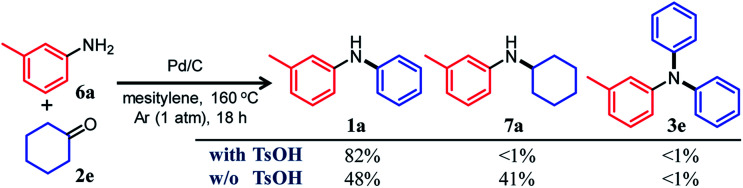
Effect of TsOH on the reaction between 3-methylaniline (**6a**) and cyclohexanone (**2e**). Reaction conditions: Pd/C (2 mol%), TsOH (0 or 10 mol%), **6a** (0.5 mmol), **2e** (0.5 mmol), mesitylene (2 mL), Ar (1 atm), temperature 160 °C, and time 18 h. Yields are relative to **6a** and were determined by GC.

In this section, we describe our attempts to develop a one-pot triarylamine synthesis from anilines and two different cyclohexanones. As discussed in the previous section, it was found that various triarylamines can be synthesized from diarylamines and cyclohexanones using a Pd/C and TsOH hybrid relay catalyst. Therefore, we first investigated whether the hybrid relay catalyst could be applied to selective diarylamine synthesis (**1a**) from 3-methylaniline (**6a**) and cyclohexanone (**2e**) under the optimized conditions for triarylamine synthesis from diarylamines and cyclohexanones. As shown in Fig. 4, in the presence of Pd/C and TsOH, an equimolar mixture of **6a** and **2e** selectively converted to the desired diarylamine **1a** in 82% yield without formation of the reductive amination product *N*-cyclohexylaniline **7a** as well as triarylamine **3e**. Under these conditions, **2e** (arylation source) was not present during the reaction because the consecutive reaction of condensation between **6a** and **2e** and disproportionation of the corresponding imine (**8e**) was completed immediately (within a few minutes) after the reaction started, as mentioned later in detail. Therefore, even if the reactions between anilines and cyclohexanones are carried out under the same conditions as used for triarylamine synthesis, diarylamines are selectively obtained without producing triarylamines because there are no arylation sources. This facilitates a very simple one-pot triarylamine synthesis, *i.e.*, after completion of the first reaction between an aniline and a cyclohexanone, it is only necessary to add a cyclohexanone as the second arylation source without isolation of the diarylamine intermediate or changing any reaction conditions, which reduces time, cost, effort, and waste generation.

The effect of TsOH on the reaction between **6a** and **2e** was crucial. When using Pd/C and TsOH, the reaction exclusively yielded **1a** after 18 h. On the other hand, in the absence of TsOH, the desired diarylamine **1a** was only obtained in 48% yield together with the formation of **7a** in 41% yield under the same conditions (Fig. 4). Therefore, in the reaction between **6a** and **2e**, TsOH likely promotes the condensation to some extent but is considered to be more important in promoting the Pd-catalyzed dehydrogenative aromatization of **7a** to **1a**, as discussed later.

**Fig. 5:**
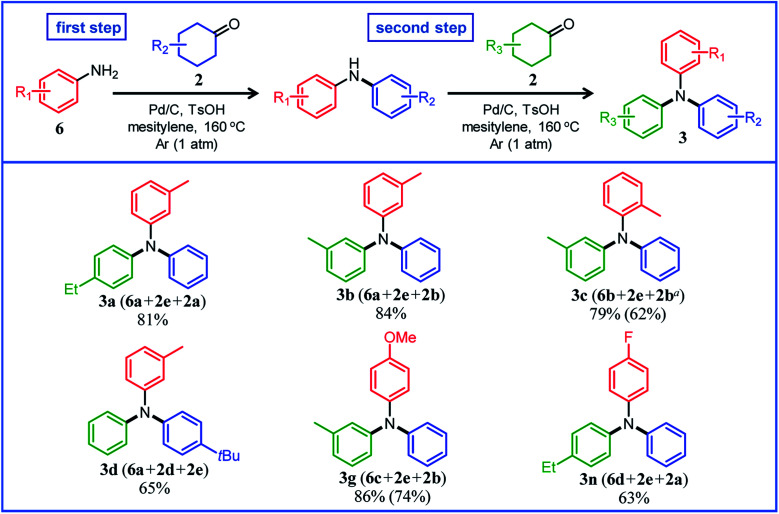
Substrate scope of the proposed one-pot triarylamine synthesis from anilines and two different cyclohexanones using a Pd/C and TsOH hybrid relay catalyst. Reaction conditions: (first step) Pd/C (2 mol%), TsOH (10 mol%), **6** (0.5 mmol), **2** (0.5 mmol), mesitylene (2 mL), Ar (1 atm), temperature 160 °C, and time 18 h. (second step) After the first step, **2** (0.55 mmol) was added to the reaction mixture, and the reaction was continued for further 2 h under the same conditions. Yields are relative to **6**. GC yields and isolated yields (values in parentheses) are shown. In all cases, phenol side products **5** were hardly detected. ^*a*^8 h for the second step.

Several results of the proposed one-pot triarylamine synthesis are summarized in Fig. 5. We successfully synthesized triarylamines with alkyl substituents at the 3- or 4-position of the phenyl rings in good yields (**3a**, **3b**, and **3d**). These triarylamines could be synthesized even if the order in which the aryl groups are introduced is changed; for example, **3a** was obtained in 65% yield using **2a** as the first arylation source (18 h) and **2e** as the second arylation source (4 h) under the conditions in Fig. 5. Regarding the synthesis of triarylamines bearing substituents at the 2-position of the phenyl rings, which was impossible in the previous section, for example, by starting from 2-methylaniline (**6b**) and sequentially introducing aryl groups into it, the desired triarylamine **3c** was synthesized in high yield using the developed one-pot synthesis. The new one-pot synthesis was also effective at introducing substituents that tend to be eliminated into the aniline substrate in advance; for example, triarylamine **3g**, which could not be successfully synthesized using 4-methoxycyclohexanone (**2g**) as the arylation source, was obtained in high yield by utilizing 4-methoxyaniline (**6c**) as the starting material and **2e** and **2b** as the arylation sources. A triarylamine bearing a fluoro group (**3n**) was also synthesized.

### Reaction pathways

**Fig. 6:**
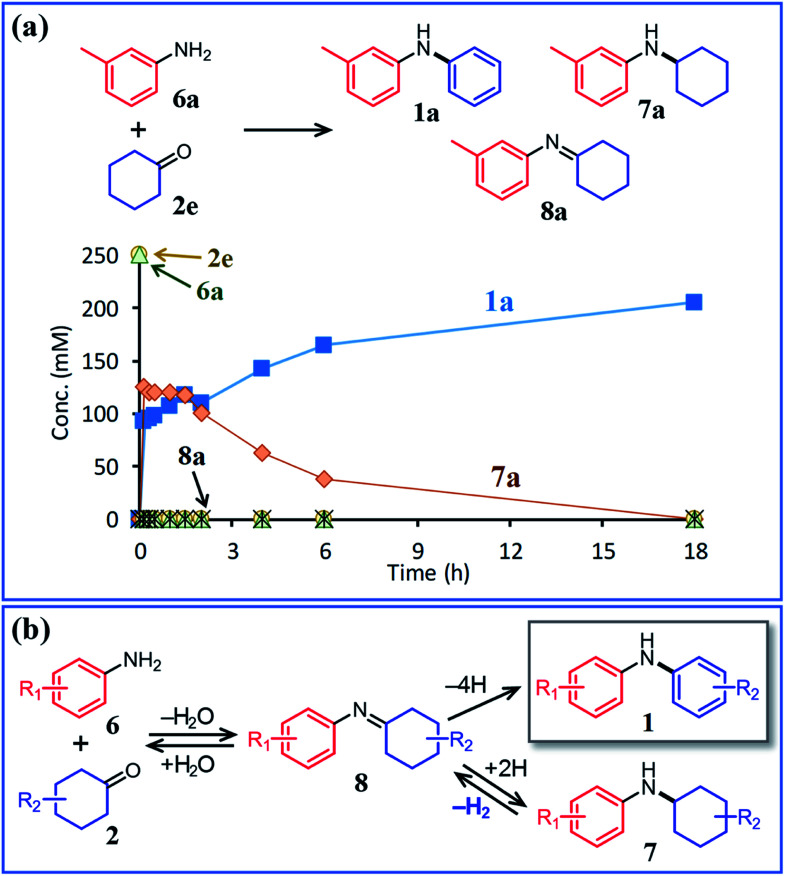
(a) Time course for the reaction between 3-methylaniline (**6a**) and cyclohexanone (**2e**) and (b) plausible reaction pathway. Reaction conditions: Pd/C (2 mol%), TsOH (10 mol%), **6a** (0.5 mmol), **2e** (0.5 mmol), mesitylene (2 mL), Ar (1 atm), and temperature 160 °C.

**Fig. 7:**
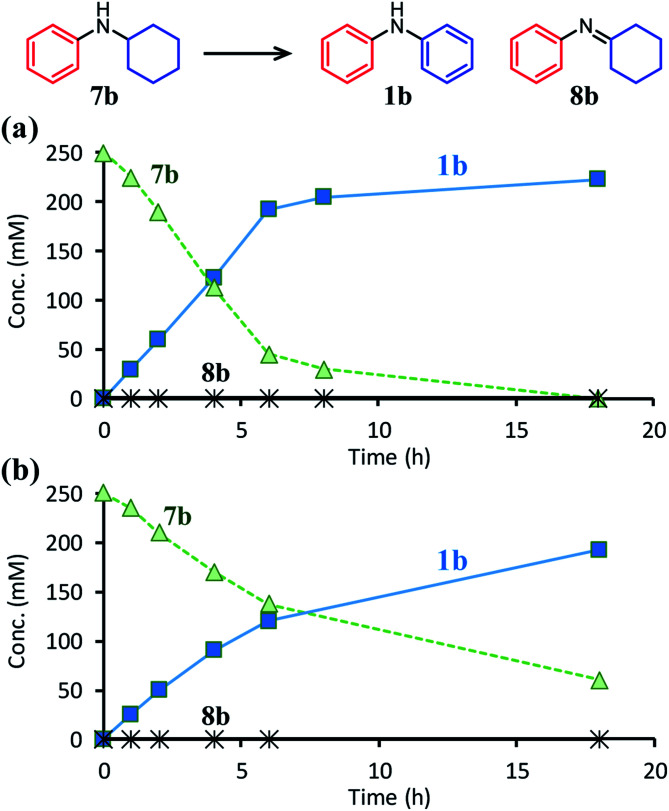
Time courses for the dehydrogenative aromatization of *N*-cyclohexylaniline (**7b**) (a) with TsOH or (b) without TsOH. Reaction conditions: Pd/C (Pd: 2 mol%), TsOH (0 or 10 mol%), **7b** (0.5 mmol), mesitylene (2 mL), temperature 160 °C, Ar (1 atm).

In this section, the reaction pathways of the proposed triarylamine synthesis are discussed in detail. To begin with, the first step involving the reaction between anilines and cyclohexanones (steps 1 and 2 in Fig. 1c) was examined. As can be seen from the time course of the reaction between **6a** and **2e** in the presence of Pd/C and TsOH (Fig. 6a), these substrates were completely consumed immediately after the start of the reaction and converted to diarylamine **1a** and *N*-cyclohexylaniline **7a**. After that, it became clear that **7a** gradually converted to **1a**. The corresponding imine (**8a**) that forms *via* the condensation of **6a** and **2e** was barely detected throughout the reaction. In a separate experiment, we confirmed that *N*-cyclohexylaniline (**7b**) was smoothly converted to diphenylamine (**1b**) in high yield under the same conditions in the presence of Pd/C and TsOH (Fig. 7a). Previously, we have reported that *N*-cyclohexylidenebenzenamine (imine **8b**) was smoothly converted to a 1 : 2 mixture of diphenylamine and *N*-cyclohexylaniline under similar acceptorless conditions in the presence of Pd-based NP catalysts.^12*a*^ Considering the present experimental results and those of our previous studies, it can be concluded that diarylamine **1a** was produced *via* the disproportionation of the imine intermediate (formed *via* the condensation of **6a** and **2e**) and that the disproportionation of **8a** was much faster than the dehydrogenation of **7a** (Fig. 6b).

The positive effect of TsOH on the dehydrogenation of **7b** to **1b** was confirmed as follows. The reaction profiles for the Pd/C-catalyzed dehydrogenation of **7b** clearly revealed that the dehydrogenation rate increased in the presence of TsOH (Fig. 7). Consequently, we conclude that TsOH promotes the rate-limiting amine dehydrogenation. In acceptorless alcohol dehydrogenation using several metal complexes, it has been reported that the activation energies of H_2_ liberation are relatively large and that the key to lowering them is to efficiently transfer H^+^ to metal hydride species.^17^ Therefore, we propose that one possible explanation for the role of TsOH on amine dehydrogenation is promoting H_2_ liberation *via* the reaction between TsOH (H^+^) and the hydride species generated on the Pd surface.

**Fig. 8:**
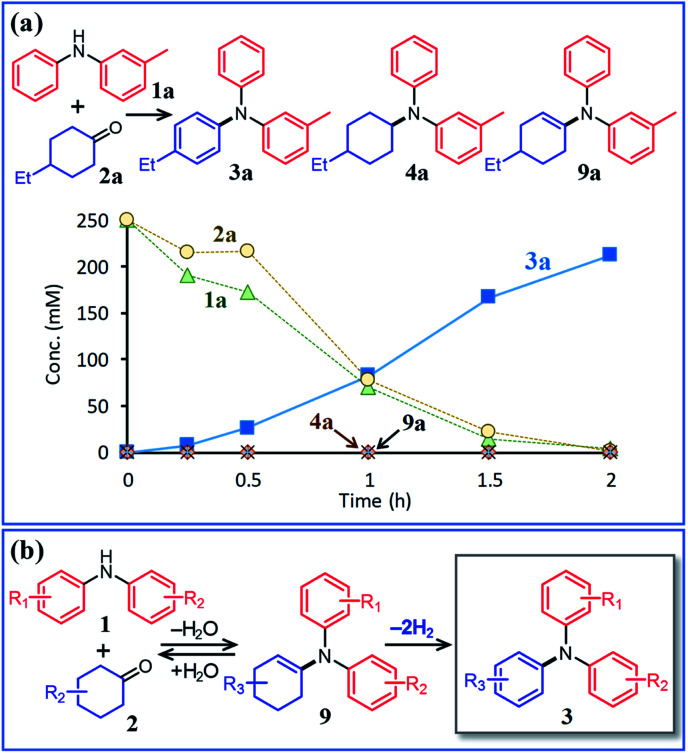
(a) Time course for the reaction between 3-methyldiphenylamine (**1a**) and 4-ethylcyclohexanone (**2a**) and (b) plausible reaction pathway. Reaction conditions: Pd/C (2 mol%), TsOH (10 mol%), **1a** (0.5 mmol), **2a** (0.5 mmol), mesitylene (2 mL), Ar (1 atm), and temperature 160 °C.

**Fig. 9:**
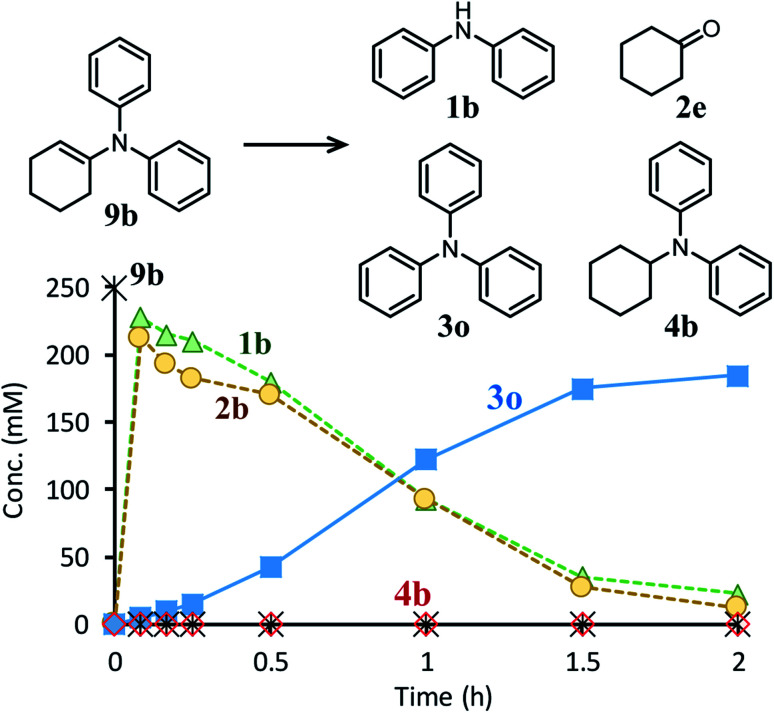
Time course for the reaction of *N*-(1-cyclohexenyl)diphenylamine (**9b**). Reaction conditions: Pd/C (2 mol%), TsOH (10 mol%), **9b** (0.4 mmol), mesitylene (1.6 mL), temperature 160 °C, Ar (1 atm).

Next, the second step of the synthesis involving the reaction between diarylamines and cyclohexanones (steps 3 and 4 in Fig. 1c) was examined. Fig. 8a shows the time course for the reaction between diarylamine **1a** and cyclohexanone **2a**. The reductive amination product **4a** and the corresponding enamine **9a** were not detected during the reaction. In addition, no traces of partially dearomatized forms of **1a** were also detected during the reaction.^18,19^ We separately synthesized *N*-cyclohexyldiphenylamine (**4b**) and *N*-(1-cyclohexenyl)diphenylamine (**9b**) and performed these reactions under the same conditions. However, the formation of triphenylamine was not observed when using **4b** as the substrate. As for the reaction of **9b**, although the hydrolysis of **9b** initially progressed to form **1b** and **2e**, triphenylamine (**3o**) was finally obtained in good yield (Fig. 9). Therefore, enamines are probably the intermediates of triarylamines in this synthesis, but it is considered that triarylamines are formed by direct dehydrogenation rather than disproportionation, unlike the reaction in the first step (Fig. 8b). Since **9a** was not observed at any time (Fig. 8a), it is presumed that the direct dehydrogenation from **9a** to **3a** is very fast. At present, although there is not enough experimental evidence, it seems possible that TsOH accelerates this dehydrogenation.

**Fig. 10:**
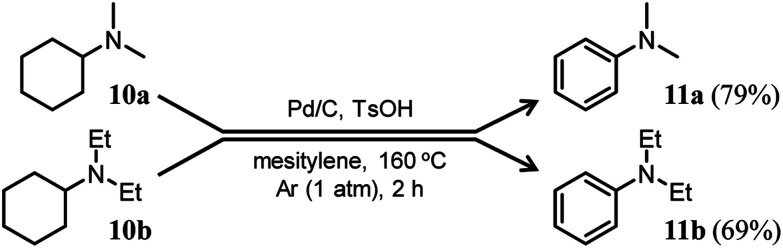
Dehydrogenative aromatization of *N*,*N*-dimethylcyclohexylamine (**10a**) and *N*,*N*-diethylcyclohexylamine (**10b**). Reaction conditions: Pd/C (2 mol%), TsOH (10 mol%), **10** (0.5 mmol), mesitylene (2 mL), Ar (1 atm), temperature 160 °C, and time 2 h. Yields are relative to **10** and determined by GC.

Here arises a question why the tertiary amine *N*-cyclohexyldiphenylamine (**4b**) did not undergo dehydrogenation while the secondary amine *N*-cyclohexylaniline (**7b**) was dehydrogenated to **1b** in Fig. 7. In the first step reaction, since imines (**8**) function as good hydrogen acceptors, it appears that diarylamines (**1**) and *N*-cyclohexylanilines (**7**) are formed by disproportionation (Fig. 6b). Thereafter, the dehydrogenations of **7** produce **8**, and eventually, **1** become the major products (Fig. 6b). In contrast, in the second step reaction, enamines (**9**) do not act as hydrogen acceptors, so it appears that triarylamines (**3**) are formed by direct dehydrogenation (Fig. 8b). We consider that this difference arises because of a steric reason; the unsaturated bond in **8** can access the Pd NP surface but that in bulkier **9** is considered difficult. The lack of progress in the dehydrogenation of **4b** is also considered to be caused by a similar steric reason. However, it may be also possible that **4b** contained two aryl groups that the lone pair in nitrogen is very well delocalized to the aryl groups, causing the Pd cannot efficiently coordinate to the nitrogen to furnish the desired dehydrogenation process. To clarify this possibility, we separately synthesized *N*,*N*-dicyclohexylaniline and performed the reaction under the conditions described in Fig. 10 using Pd/C and TsOH. The reaction progressed marginally, affording **4b** in 14% yield. In this case, triphenylamine was not detected. Therefore, it may be concluded that the reason for no reaction of **4b** is due to not only the steric reason but also the effect of its lone pair delocalization. We also found during this work that when the Pd/C and TsOH hybrid relay catalyst was used, the dehydrogenative aromatization of several tertiary amines, such as *N*,*N*-dimethylcyclohexylamine (**10a**) and *N*,*N*-diethylcyclohexylamine (**10b**), which are less sterically crowded around the N positions or smaller delocalization effect than *N*-cyclohexyldiphenylamine and *N*,*N*-dicyclohexylaniline, proceeded effectively, giving the corresponding anilines (Fig. 10). To the best of our knowledge, this is the first report of dehydrogenative aromatization of tertiary amines.

## Conclusion

We have successfully developed efficient catalytic procedures for synthesizing triarylamines *via* acceptorless dehydrogenative aromatization. In the presence of a Pd/C and TsOH hybrid relay catalyst, various types of structurally diverse unsymmetrically substituted triarylamines could be synthesized starting from diarylamines and cyclohexanones. Moreover, a one-pot sequential triarylamine synthesis from anilines and two different cyclohexanones was also successful. Owing to their practical and environmentally benign nature, we hope that the catalytic transformations developed in this study will find wide applications in the synthesis of arylamine derivatives and related compounds. The discovery that this type of dehydrogenation is promoted in the presence of a suitable Brønsted acid co-catalyst is also very interesting and can be expected to apply to a variety of related functional transformations, which may be exploited in future synthetic schemes.

## Experimental section

### Instruments and reagents

Gas chromatography (GC) analyses were performed using a Shimadzu GC-2014 equipped with a flame ionization detector using an InertCap5 capillary column. GC-MS spectra were recorded using a Shimadzu GCMS-QP2010 equipped with an InertCap5 capillary column at an ionization voltage of 70 eV. Liquid-state nuclear magnetic resonance (NMR) spectra were recorded using a JEOL JNM-ECA-500. ^1^H and ^13^C NMR spectra were measured at 500.2 and 125.8 MHz, respectively, using tetramethylsilane as an internal standard (*δ* = 0 ppm). ICP-AES analyses were performed using a Shimadzu ICPS-8100. Transmission electron microscopy observations were performed using a JEOL JEM-2000EX. Column chromatography on silica gel was performed using a Biotage Isolera system. Elemental analyses for C, H, and N were performed using an Elementar vario MICRO cube. Pd/C (lot. no. 217-024030, 217-172450, N.E. CHEMCAT), Ru/C (lot. no. 417-020160, N.E. CHEMCAT), Rh/C (lot. no. MCM7367, FUJIFILM Wako Pure Chemical), and Pt/C (lot. no. 117-061210, N.E. CHEMCAT) were commercially available. Al_2_O_3_ (KHS-24, Sumitomo Chemical), TiO_2_ (ST-01, Ishihara Sangyo Kaisya), CeO_2_ (544841-25G, Aldrich), ZrO_2_ (37022, Nacalai Tesque), and Mg_6_Al_2_(OH)_16_CO_3_·4H_2_O (LDH, Tomita Pharmaceutical Co., Ltd.) were commercially available. Pd/support catalysts were prepared according to a literature procedure.^20^ Substrates (except for **4b** and **9b**) and solvents were obtained from TCI, Aldrich, Kanto Chemical, FUJIFILM Wako Pure Chemical (reagent grade), or Combi-blocks, and purified prior to use, if necessary.^21^ Compounds **4b** and **9b** were synthesized according to a literature procedure.^22^*N*,*N*-Dicyclohexylaniline was synthesized according to a literature procedure.^23^

### Catalytic reactions

Catalytic reactions were typically carried out according to the following procedure. Pd/C (2 mol%), TsOH (10 mol%), diarylamine (**1**, 0.5 mmol), cyclohexanone (**2**, 0.5 mmol), *n*-hexadecane (0.1 mmol, internal standard), mesitylene (2 mL), and a Teflon-coated magnetic stir bar were successively placed into a ∼20 mL Schlenk flask reactor, and then the mixture was stirred at 160 °C under Ar (1 atm). Substrate conversions and product yields were periodically monitored by GC analysis. For the isolation of the products, the internal standard was not used. After the reaction, the catalyst was removed by simple filtration and the filtrate was concentrated by evaporation of the mesitylene solvent. The crude product was subjected to column chromatography on silica gel (typically using hexane and toluene as an eluent), yielding the pure triarylamine product. The products were identified by GC-MS, NMR (^1^H and ^13^C), and/or elemental analyses. Detection of H_2_ in the gas phase was carried out by GC-MS analysis. Quantification of the H_2_ formed was achieved by measurement of the volume of evolved gas.

## Conflicts of interest

There are no conflicts to declare.

## Supplementary Material

SC-011-C9SC06442G-s001
