# Supplementary material for: Synthesis of unsymmetrically substituted triarylamines via acceptorless dehydrogenative aromatization using a Pd/C and p-toluenesulfonic acid hybrid relay catalyst
Source: Chem Sci. 2020 Mar 25;11(16):4074–84. doi: 10.1039/c9sc06442g (PMC8152582; doi:10.1039/c9sc06442g)
Supplement: SC-011-C9SC06442G-s001 [file SC-011-C9SC06442G-s001.pdf]

Electronic Supplementary Material (ESI) for Chemical Science.
This journal is © The Royal Society of Chemistry 2020

Electronic Supplementary Information (ESI) for

Synthesis of unsymmetrically substituted triarylamines *via* acceptorless dehydrogenative aromatization using a Pd/C and *p*-toluenesulfonic acid hybrid relay catalyst

Satoshi Takayama,[a] Takafumi Yatabe,[a] Yu Koizumi,[a] Xiongjie Jin,[b] Kyoko Nozaki,[b] Noritaka Mizuno[a] and Kazuya Yamaguchi*[a]

[a]*Department of Applied Chemistry, School of Engineering, The University of Tokyo, 7-3-1 Hongo, Bunkyo-ku, Tokyo 113-8656, Japan. E-mail: kyama@appchem.t.u-tokyo.ac.jp; Fax: +81-3-5841-7220*

[b]*Department of Chemistry and Biotechnology, School of Engineering, The University of Tokyo, 7-3-1 Hongo, Bunkyo-ku, Tokyo 113-8656, Japan.*

Table of Contents

▪ TEM images and particle size distributions (Fig. S1) page S1
▪ Pd contents and average particle sizes (Table S1) page S2
▪ Effect of solvents (Table S2) page S2
▪ Effect of temperatures (Table S3) page S2
▪ Effect of removal of Pd/C (verification of heterogeneous catalysis) (Fig. S2) page S3
▪ Reuse test (Fig. S3) page S3
▪ Data of substrates pages S4
▪ Data of triarylamines pages S4–S8
▪ Additional references page S8
▪ NMR spectra pages S9–S22

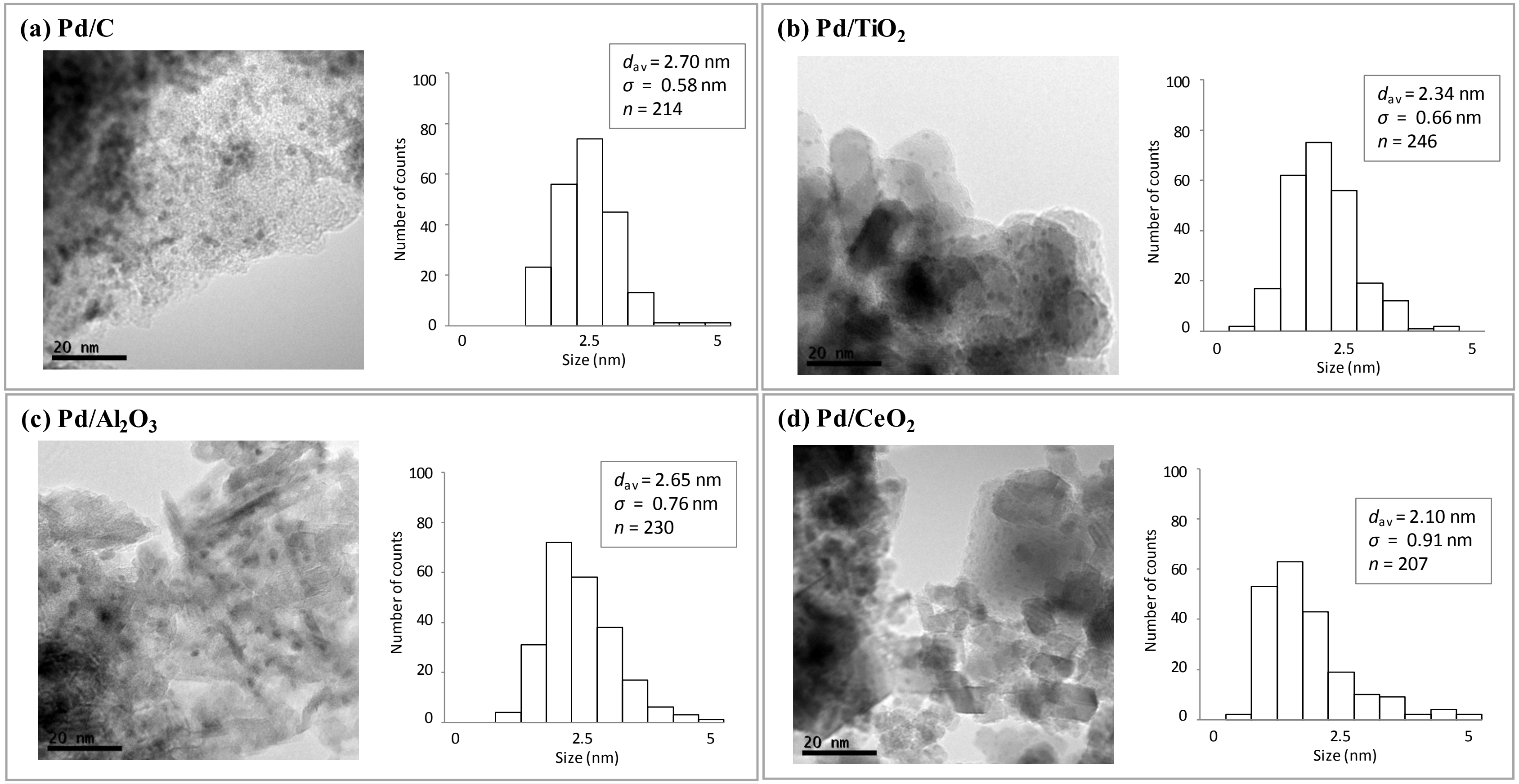

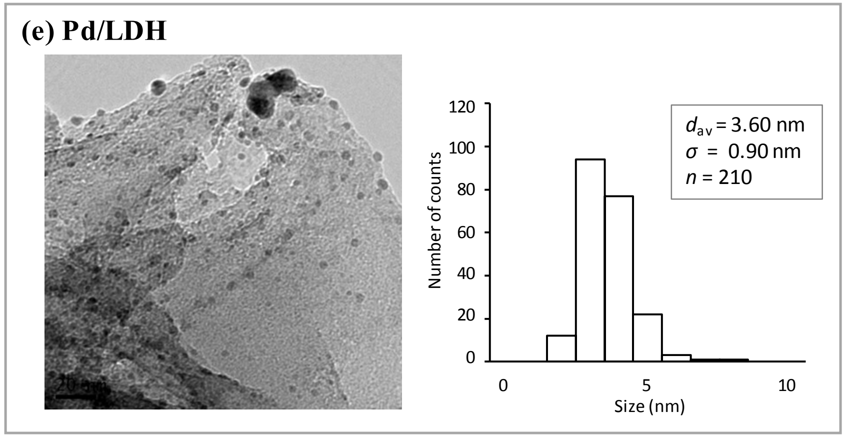

Fig. S1 TEM images and particle size distributions. (a) Pd/C (average particle size (d_{av}) = 2.70 nm; standard deviation (σ) = 0.58 nm; total number of counts (n) = 214), (b) Pd/TiO$_2$ (d_{av} =2.34 nm; σ = 0.66 nm; n = 246), (c) Pd/Al$_2$O$_3$ (d_{av} = 2.65 nm; σ = 0.76 nm; n = 230), (d) Pd/CeO$_2$ (d_{av} = 2.10 nm; σ = 0.91 nm; n = 207), (e) Pd/LDH (d_{av} = 3.60 nm; σ = 0.9 nm; n = 210).

Table S1 Pd contents and average particle sizes

Catalyst	Pd content (mmol g^{-1})	Average particle size (nm)
Pd/C	0.226	2.70
Pd/TiO_2	0.222	2.34
Pd/Al_2O_3	0.227	2.65
Pd/CeO_2	0.209	2.10
Pd/LDH	0.234	3.60

Table S2 Effect of solvents[a]

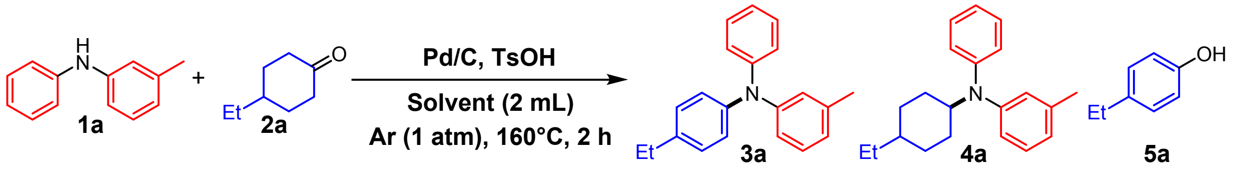

Entry	Solvent	Yield (%)		
		**3a**	**4a**	**5a**
**1**	**Mesitylene**	**85**	**<1**	**<1**
2	Decane	72	<1	<1
3	Diglyme	68	<1	<1
4	DMA	11	<1	2
5	NMP	5	<1	5

[a]Reaction conditions: Pd/C (Pd: 2 mol%), TsOH (10 mol%), **1a** (0.5 mmol), **2a** (0.5 mmol), solvent (2 mL), Ar (1 atm), 160°C, 2 h. Yields were determined by GC analysis using *n*-hexadecane as an internal standard.

Table S3 Effect of temperatures[a]

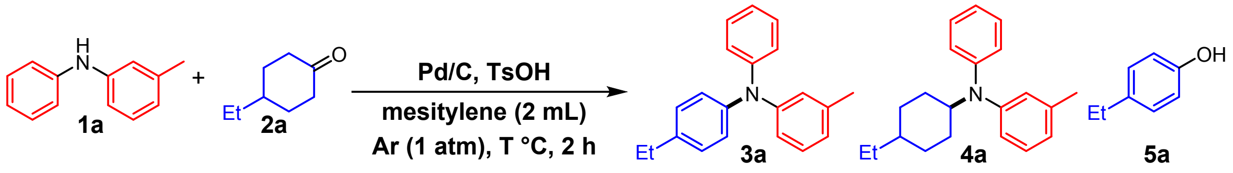

Entry	Temperature [°C]	Yield (%)		
		**3a**	**4a**	**5a**
**1**	**160**	**85**	**<1**	**<1**
2	150	64	<1	1
3	140	21	<1	1
4	130	7	<1	1

[a]Reaction conditions: Pd/C (Pd: 2 mol%), TsOH (10 mol%), **1a** (0.5 mmol), **2a** (0.5 mmol), mesitylene (2 mL), Ar (1 atm), 2 h. Yields were determined by GC analysis using *n*-hexadecane as an internal standard.

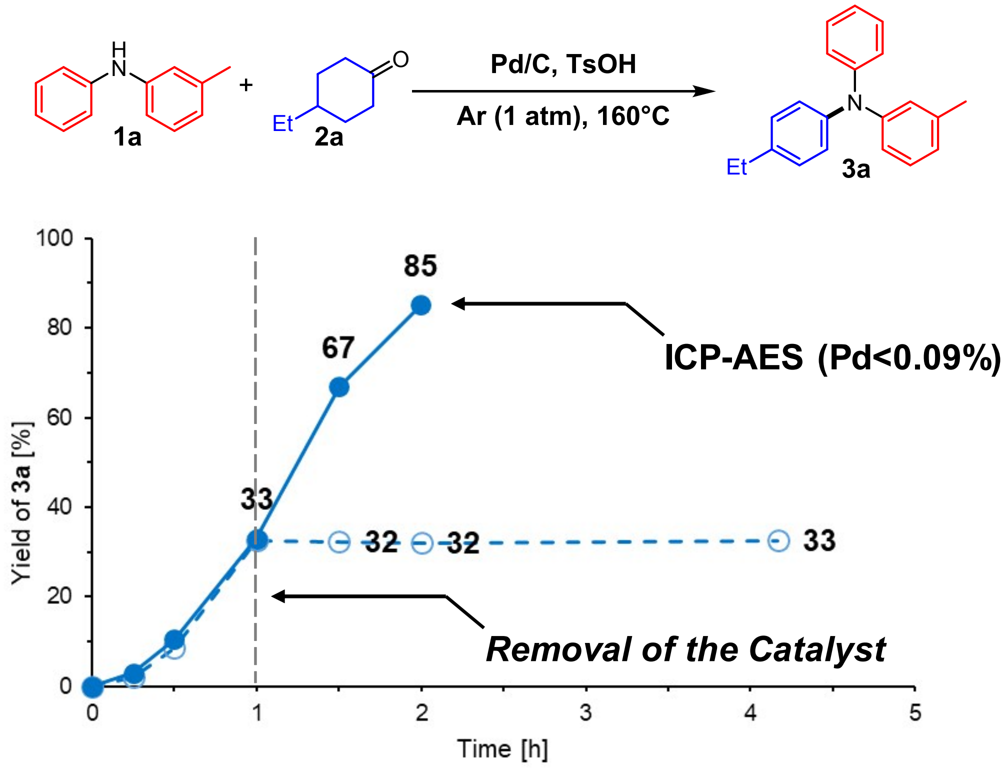

Fig. S2 Effect of removal of Pd/C (verification of heterogeneous catalysis). Reaction conditions: Pd/C (Pd: 2 mol%), TsOH (10 mol%), **1a** (0.5 mmol), **2a** (0.5 mmol), mesitylene (2 mL), 160°C, Ar (1 atm). Yields were determined by GC analysis using *n*-hexadecane as an internal standard. The arrow indicates the removal of Pd/C by hot filtration.

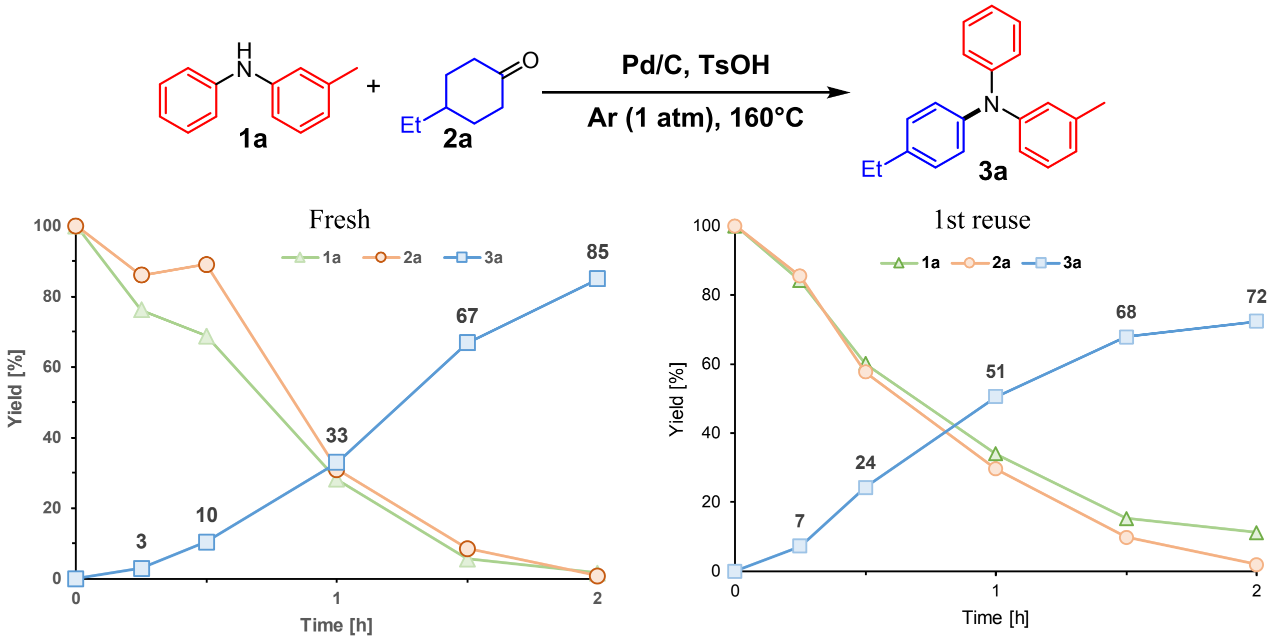

Fig. S3 Reuse test. Reaction conditions: Pd/C (Pd: 2 mol%), TsOH (10 mol%), **1a** (0.5 mmol), **2a** (0.5 mmol), mesitylene (2 mL), 160°C, Ar (1 atm). Yields were determined by GC analysis using *n*-hexadecane as an internal standard.

Data of substrates

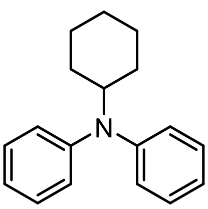

***N*-cyclohexyldiphenylamine (4b)** (CAS No. 4705-13-9): Isolated as yellow powders (Eluent: hexane/toluene= 1/1, R_f = 0.75). ^{1}H NMR (500 MHz, $CDCl_3$, TMS): δ 7.42–7.23 (m, 4H), 6.99–6.95 (m, 2H), 6.84–6.81 (m, 4H), 3.82 (tt, J = 11.6, 3.4 Hz, 1H, CH), 2.03–2.00 (m, 2H), 1.80–1.76 (m, 2H), 1.63–1.60 (m, 1H), 1.43–1.34 (m, 2H), 1.15–1.07 (m, 2H), 1.04–0.95 (m, 1H). ^{13}C{^{1}H} NMR (125MHz, $CDCl_3$, TMS): δ 146.3, 129.1, 122.7, 121.4, 56.6, 31.7, 26.2, 25.7. MS (70 eV, EI): *m*/*z* (%): 252 (11), 251 (52) [M^+], 209 (17), 208 (100), 194 (8), 193 (6), 182 (5), 169 (21), 168 (14), 167 (12), 104 (13), 91 (8), 77 (17), 55 (8), 51 (6).

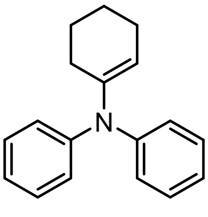

***N*-(1-cyclohexenyl)diphenylamine (9b)**: Isolated as colorless crystals (Eluent: hexane/toluene = 95/5, R_f = 0.56). ^{1}H NMR (500 MHz, $CDCl_3$, TMS): δ 7.20–7.16 (m, 4H), 7.04–7.02 (m, 4H), 6.91–6.88 (m, 2H), 5.50 (m, 2H), 2.11 (m, 4H), 1.72–1.62 (m, 4H). ^{13}C{^{1}H} NMR (125MHz, $CDCl_3$, TMS): δ 147.3, 143.1, 128.8, 122.5, 122.0, 121.3, 27.8, 25.0, 23.2, 22.3. MS (70 eV, EI): *m*/*z* (%): 250 (16), 249 (89) [M^+], 248 (100), 221 (8), 220 (31), 206 (19), 204 (6), 168 (6), 167 (12), 158 (6), 157 (8), 131 (6), 130 (55), 129 (8), 128 (5), 119 (36), 118 (14), 117 (17), 115 (9), 104 (9), 103 (6), 91 (13), 78 (6), 77 (54), 65 (5), 51 (19).

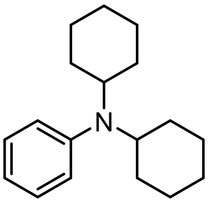

***N,N*-dicyclohexylaniline:** (CAS No. 63302-13-6): Isolated as colorless crystals (Eluent: hexane/toluene = 9/1, R_f = 0.04). ^{1}H NMR (500 MHz, $CDCl_3$, TMS): δ 7.22–7.12 (m, 2H), 6.98–6.90 (m, 2H), 6.84–6.75 (m, 1H), 3.29–3.18 (m, 2H), 1.79–1.43 (m, 14H), 1.37–1.06 (m, 6H). ^{13}C{^{1}H} NMR (125MHz, $CDCl_3$, TMS): δ 148.5, 128.2, 121.1, 119.0, 57.5, 31.9, 26.3, 26.0. MS (70 eV, EI): *m*/*z* (%): 258 (7), 257 (35) [M^+], 215 (16), 214 (100), 175 (6), 174 (6), 133 (9), 132 (81), 130 (8), 120 (7), 119 (63), 118 (14), 117 (8), 106 (8), 104 (16), 91 (7), 83 (5), 77 (22), 55 (28).

Data of triarylamines

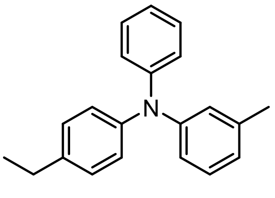

***N*-(4-ethylphenyl)-3-methyl-*N*-phenylaniline (3a):** 85% GC yield, 70% isolated yield (Fig. 3). Isolated as colorless crystals (Eluent: hexane/toluene = 19/1, R_f = 0.27). ^{1}H NMR (500 MHz, $CDCl_3$, TMS): δ 7.20–7.17 (m, 2H, Ar), 7.10–7.04 (m, 5H, Ar), 7.01–6.99 (m, 2H, Ar), 6.95–6.92 (m, 1H, Ar), 6.90 (s, 1H, Ar), 6.87–6.85 (m, 1H, Ar), 6.79–6.77 (m, 1H, Ar), 2.59 (q, J = 7.6 Hz, 2H, CH_2), 1.22 (t, J = 7.6 Hz, 3H, CH_3). ^{13}C{^{1}H} NMR (125MHz, $CDCl_3$, TMS): δ 148.1, 147.9, 145.4, 138.9, 138.8, 129.0, 128.9. 128.6, 124.7, 124.5, 123.5, 123.2, 122.0, 121.1, 28.2, 21.4, 15.5. MS (70 eV, EI): *m*/*z* (%): 288 (20), 287 (89) [M^+], 273 (23), 272 (100), 258 (6), 257 (5), 256 (7), 180 (8), 167 (6), 154 (6), 153 (6), 128 (7), 127 (5), 115 (5), 91 (6), 77 (10), 65 (6), 51 (5). Anal. Calc. for $C_{21}H_{21}N$: C, 87.76; H, 7.37; N, 4.87. Found: C, 87.84; H, 7.61; N, 4.76.

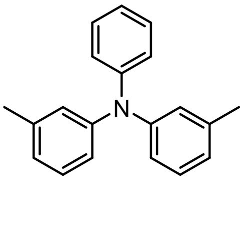

3-methyl-*N*-phenyl-*N*-(*m*-tolyl) aniline (3b) (CAS No. 13511-11-0)[S1]: 80% GC yield, 70% isolated yield (Fig. 3). Isolated as white powder (Eluent: hexane/toluene = 23/2, R_f

= 0.43). ^{1}H NMR (500 MHz, $CDCl_3$, TMS): δ 7.22–7.19 (m, 2H, Ar), 7.12–7.09 (m, 2H, Ar), 7.06–7.05 (m, 2H, Ar), 6.98–6.94 (m, 1H, Ar), 6.90 (s, 2H, Ar), 6.88–6.86 (m, 2H, Ar), 6.82–6.80 (m, 2H, Ar), 2.24 (s, 6H, CH_3). $^{13}C\{^{1}H\}$ NMR (125MHz, $CDCl_3$, TMS): δ 148.0, 147.8, 139.0, 129.1, 128.9, 124.9, 123.9, 123.5, 122.3, 121.4, 21.4. These NMR spectral data accord with those previously reported.[S1] MS (70 eV, EI): *m*/*z* (%): 274 (22), 273 (100) [M^{+}], 272 (19), 258 (12), 257 (22), 256 (6), 243 (5), 180 (10), 167 (7), 166 (5), 155 (5), 136 (5), 128 (7), 127 (5), 115 (6), 91 (6), 77 (9), 65 (9), 51 (5).

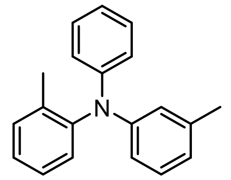

2-methyl-*N*-phenyl-*N*-(*m*-tolyl) aniline (3c) (CAS No. 1648726-23-1): 79% GC yield, 62% isolated yield (Fig. 4). Isolated as colorless crystals (Eluent: hexane/toluene = 97/3, R_f = 0.43). ^{1}H NMR (500 MHz, $CDCl_3$, TMS): δ 7.22–7.04 (m, 7H, Ar), 6.95–6.93 (m, 2H, Ar), 6.89–6.86 (m, 1H, Ar), 6.80–6.71 (m, 3H, Ar), 2.21 (s, 3H, CH_3), 2.03 (s, 3H, CH_3). $^{13}C\{^{1}H\}$ NMR (125MHz, $CDCl_3$, TMS): δ 147.6, 147.4, 145.4, 138.7, 136.4, 131.6, 129.6, 128.9, 128.8, 127.3, 125.8, 122.3, 122.2, 121.4, 121.1, 118.9, 21.5, 18.6. MS (70 eV, EI): *m*/*z* (%): 274 (21), 273 (100) [M^{+}], 272 (12), 258 (16), 257 (8), 256 (7), 243 (9), 196 (7), 194 (5), 182 (11), 181 (8), 180 (26), 167 (10), 166 (7), 165 (6), 137 (5), 136 (6), 128 (6), 115 (5), 91 (6), 77 (10), 65 (10), 51(7).

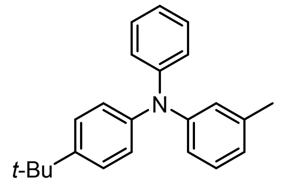

***N*-(4-(tert-butyl) phenyl)-3-methyl-*N*-phenylaniline (3d)**: 73% GC yield, 76% isolated yield. Isolated as colorless crystals (Eluent: hexane/toluene = 19/1, R_f = 0.35). ^{1}H NMR (500 MHz, $CDCl_3$, TMS): δ 7.24–7.18 (m, 4H, Ar), 7.11–7.08 (m, 1H, Ar), 7.06–7.05 (m, 2H, Ar), 7.00–6.99 (m, 2H, Ar), 6.96–6.93 (m, 1H, Ar), 6.91 (s, 1H, Ar), 6.87–6.86 (m, 1H, Ar), 6.80–6.78 (m, 1H, Ar), 2.23 (s, 3H, CH_3), 1.30 (s, 9H, *t*-Bu). $^{13}C\{^{1}H\}$ NMR (125MHz, $CDCl_3$, TMS): δ 148.1, 147.9, 145.5, 145.1, 138.9, 129.0, 128.9, 126.0, 124.6, 123.8, 123.7, 123.3, 122.1, 121.2, 34.2, 31.4, 21.4. MS (70 eV, EI): *m*/*z* (%): 316 (14), 315 (52) [M^{+}], 301 (24), 300 (100), 285 (10), 167 (12), 150 (5), 136 (6), 135 (5), 128 (13), 77 (6). Anal. Calc. for $C_{23}H_{25}N$: C, 87.57; H, 7.99; N, 4.44. Found: C, 87.17; H, 7.92; N, 4.28.

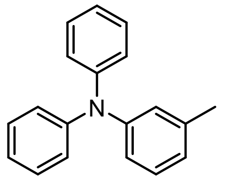

3-methyl-*N*, *N*-diphenylaniline (3e) (CAS No. 4316-54-5)[S2]: 78% GC yield, 66% isolated yield (Fig. 3). Isolated as white powder (Eluent: hexane/toluene =19/1, R_f = 0.36). ^{1}H NMR (500 MHz, $CDCl_3$, TMS): δ 7.23–7.20 (m, 4H, Ar), 7.13–7.10 (m, 1H, Ar), 7.07–7.06 (m, 4H), 6.99–6.96 (m, 2H, Ar), 6.91 (s, 1H, Ar), 6.89–6.87 (m, 1H, Ar), 6.83–6.81 (m, 1H, Ar), 2.24 (s, 3H, CH_3). $^{13}C\{^{1}H\}$ NMR (125MHz, $CDCl_3$, TMS): δ 147.9, 147.7, 139.0, 129.1, 129.0, 125.0, 124.0, 123.7, 122.5, 121.5, 21.4. These NMR spectral data accord with those previously reported.[S2] MS (70 eV, EI): *m*/*z* (%): 260 (21), 259 (100) [M^{+}], 258 (25), 244 (12), 243 (21), 242 (5), 180 (7), 167 (14), 166 (11), 141 (8), 129 (5), 128 (7), 115 (8), 77 (14), 65 (7), 51 (11).

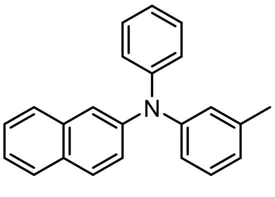

***N*-phenyl-*N*-(*m*-tolyl) naphthalen-2-amine (3f)**: 84% GC yield, 84% isolated yield (Fig. 3). Isolated as colorless crystals (Eluent: hexane/toluene = 19/1, R_f = 0.34). ^{1}H NMR (500 MHz, $CDCl_3$, TMS): δ 7.72–7.70 (m, 1H, Ar), 7.68–7.66 (m, 1H, Ar),

7.55–7.53 (m, 1H, Ar), 7.41–7.40 (m, 1H, Ar), 7.36–7.21 (m, 5H, Ar), 7.14–7.10 (m, 3H, Ar), 7.01–6.98 (m, 1H, Ar), 6.95 (s, 1H, Ar), 6.93–6.92 (m, 1H, Ar), 6.85–6.83 (m, 1H, Ar), 2.24 (s, 3H, CH_3). $^{13}C\{^1H\}$ NMR (125MHz, $CDCl_3$, TMS): δ 147.9, 147.7, 145.6, 139.1, 134.4, 129.9, 129.2, 128.7, 127.5, 126.9, 126.2, 125.2, 125.1, 124.3, 123.8, 122.8, 122.6, 121.8, 121.6, 120.0, 21.4. MS (70 eV, EI): *m*/*z* (%): 310 (25), 309 (100) [M^+], 308 (22), 294 (7), 293 (11), 217 (9), 216 (7), 192 (6), 191 (8), 155 (6), 146 (5), 115 (5), 77 (5). Anal. Calc. for $C_{23}H_{19}N{\cdot}0.3\ H_2O$: C, 87.75; H, 6.28; N, 4.45. Found: C, 87.87; H, 6.41; N, 4.48.

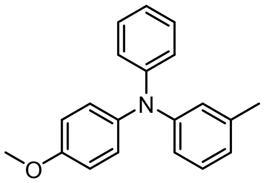

***N*-(4-methoxyphenyl)-3-methyl-*N*-phenylaniline (3g)** (CAS No. 1648726-90-2): 86% GC yield, 74% isolated yield (Fig. 4). Isolated as colorless crystals (Eluent: hexane/toluene = 4/1, R_f = 0.33). ^1H NMR (500 MHz, $CDCl_3$, TMS): δ 7.18–7.15 (m, 2H, Ar), 7.09–7.00 (m, 5H, Ar), 6.92–6.74 (m, 6H, Ar), 3.76 (s, 3H, CH_3 methoxy), 2.22 (s, 3H, CH_3). $^{13}C\{^1H\}$ NMR (125MHz, $CDCl_3$, TMS): δ 156.0, 148.2, 148.0, 140.8, 138.8, 129.0, 128.8, 127.2, 123.6, 122.8, 122.7, 121.6, 120.2, 114.6, 55.3, 21.4. MS (70 eV, EI): *m*/*z* (%): 290 (23), 289 (100) [M^+], 275 (21), 274 (93), 230 (6), 145 (6), 128 (9), 91 (5), 77 (7), 65 (5).

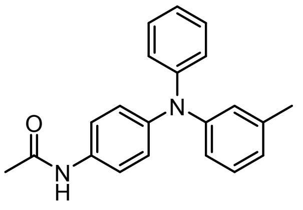

***N*-(4-(phenyl (*m*-tolyl) amino) phenyl) acetamide (3h)**: 31% GC yield, 32% isolated yield (Fig. 3). Isolated as white powder (Eluent: hexane/EtOAc = 2/3, R_f = 0.37). ^1H NMR (500 MHz, $CDCl_3$, TMS): δ 7.61 (s, 1H, NH), 7.38–7.35 (m, 2H, Ar), 7.22–7.19 (m, 2H, Ar), 7.12–7.09 (m, 1H, Ar), 7.03–7.01 (m, 4H, Ar), 6.98–6.95 (m, 1H, Ar), 6.87–6.80 (m, 3H, Ar), 2.23 (s, 3H, CH_3), 2.14 (s, 3H, CH_3 amide). $^{13}C\{^1H\}$ NMR (125MHz, $CDCl_3$, TMS): δ 168.4 (CO), 147.8, 147.6, 144.2, 139.0, 132.8, 129.1, 129.0, 124.8, 124.5, 123.6, 123.5, 122.3, 121.2, 121.1, 24.3, 21.4. MS (70 eV, EI): *m*/*z* (%): 317 (24), 316 (100) [M^+], 275 (15), 274 (52), 273 (59), 182 (5), 181 (5), 167 (5), 129 (6), 128 (9), 91 (5), 77 (7), 65 (6). Anal. Calc. for $C_{21}H_{20}N_2O{\cdot}0.7\ H_2O$: C, 76.66; H, 6.56; N, 8.51. Found: C, 76.42; H, 6.14; N, 8.42.

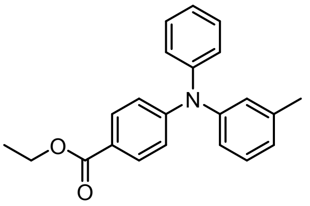

ethyl 4-(phenyl (*m*-tolyl) amino) benzoate (3i) (CAS No. 1344716-76-2)[S3]: 54% GC yield, 58% isolated yield (Fig. 3). Isolated as colorless crystals (Eluent: hexane/toluene =1/4, R_f = 0.39). ^1H NMR (500 MHz, $CDCl_3$, TMS): δ 7.88–7.87 (m, 2H, Ar), 7.32–7.29 (m, 2H, Ar), 7.21–7.18 (m, 1H, Ar), 7.16–7.10 (m, 3H, Ar), 7.00–6.94 (m, 5H, Ar), 4.33 (q, J = 7.2 Hz, 2H, CH_2), 2.29 (s, 3H, CH_3), 1.36 (t, J = 7.2 Hz, 3H, CH_3), $^{13}C\{^1H\}$ NMR (125MHz, $CDCl_3$, TMS): δ 166.4 (CO), 152.0, 146.7, 146.6, 139.4, 130.7, 129.4, 129.3, 126.4, 125.6, 125.3, 124.2, 123.0, 122.3, 120.0, 60.4, 21.2, 14.4. MS (70 eV, EI): *m*/*z* (%): 332 (24), 331(100) [M^+], 304 (9), 303 (42), 302 (5), 286 (18), 259 (5), 258 (15), 257 (9), 256 (7), 244 (5), 243 (16), 242 (8), 241 (6), 180 (5), 167 (9), 166 (8), 143 (6), 115 (5), 77 (7), 65 (6).

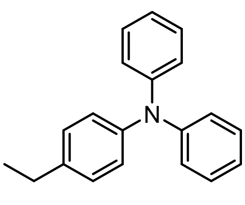

4-ethyl-*N*, *N*-diphenylaniline (3j) (CAS No. 36809-22-0)[S4]: 67% GC yield, 63% isolated yield (Fig. 3). Isolated as colorless crystals (Eluent: hexane/toluene =19/1, R_f = 0.39). ^1H NMR (500 MHz, $CDCl_3$, TMS): δ 7.22–7.18 (m, 4H, Ar), 7.08–7.00 (m, 8H,

Ar), 6.96–6.93 (m, 2H, Ar), 2.60 (q, J = 7.6 Hz, 2H, CH_2), 1.22 (t, J = 7.6 Hz, 3H, CH_3). $^{13}C\{^1H\}$ NMR (125MHz, $CDCl_3$, TMS): δ 148.0, 145.4, 139.0, 129.1, 128.6, 124.8, 123.6, 122.2, 28.2, 15.5. These NMR spectral data accord with those previously reported.[S4] MS (70 eV, EI): m/z (%): 324 (27), 323 (100) [M^+], 309 (19), 308 (74), 294 (9), 293 (7), 292 (5), 230 (7), 217 (8), 216 (7), 136 (5), 77 (6).

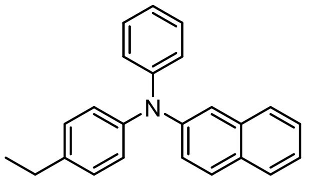

***N*-(4-ethylphenyl)-*N*-phenylnaphthalen-2-amine (3k):** 73% GC yield, 76% isolated yield (Fig. 3). Isolated as colorless crystals (Eluent: hexane/toluene =19/1, R_f = 0.32). ^1H NMR (500 MHz, $CDCl_3$, TMS): δ 7.70–7.69 (m, 1H, Ar), 7.66–7.64 (m, 1H, Ar), 7.53–7.52 (m, 1H, Ar), 7.39–7.39 (m, 1H, Ar), 7.34–7.19 (m, 5H, Ar), 7.15–7.04 (m, 6H, Ar), 6.99–6.96 (m, 1H, Ar), 2.60 (q, J = 7.6 Hz, 2H, CH_2), 1.23 (t, J = 7.6 Hz, 3H, CH_3). $^{13}C\{^1H\}$ NMR (125MHz, $CDCl_3$, TMS): δ 147.9, 145.6, 145.3, 139.2, 134.4, 129.8, 129.2, 128.7, 127.5, 126.8, 126.2, 124.9, 124.2, 124.2, 123.9, 122.5, 119.5, 28.2, 15.5. MS (70 eV, EI): m/z (%): 324 (27), 323 (100) [M^+], 322 (5), 309 (17), 308 (69), 294 (7), 293 (6), 230 (5), 217 (6), 127 (5), 115 (5), 77 (6). Anal. Calc. for $C_{24}H_{21}N\cdot0.3\ H_2O$: C, 87.86; H, 6.62; N, 4.26. Found: C, 87.76; H, 6.48; N, 4.29.

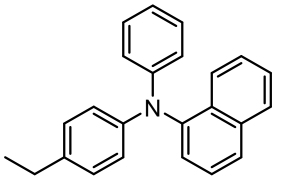

***N*-(4-ethylphenyl)-*N*-phenylnaphthalen-1-amine (3l):** 62% GC yield, 62% isolated yield. Isolated as colorless crystals (Eluent: hexane/toluene =19/1, R_f = 0.34). ^1H NMR (500 MHz, $CDCl_3$, TMS): δ 7.95–7.94 (m, 1H, Ar), 7.85–7.83 (m, 1H, Ar), 7.72–7.71 (m, 1H, Ar), 7.43–7.39 (m, 2H, Ar), 7.33–7.29 (m, 2H, Ar), 7.15–7.12 (m, 2H, Ar), 7.02–6.94 (m, 6H, Ar), 6.87–6.84 (m, 1H, Ar), 2.56 (q, J = 7.6 Hz, 2H, CH_2), 1.19 (t, J = 7.6 Hz, 3H, CH_3). $^{13}C\{^1H\}$ NMR (125MHz, $CDCl_3$, TMS): δ 148.8, 146.0, 143.7, 137.9, 135.2, 131.3, 128.9, 128.5, 128.3, 127.1, 126.3, 126.24, 126.18, 126.0, 124.3, 122.5, 121.0, 120.9, 28.1, 15.5. MS (70 eV, EI): m/z (%): 324 (27), 323 (100) [M^+], 309 (19), 308 (74), 294 (9), 293 (7), 292 (5), 230 (7), 217 (8), 216 (7), 136 (5), 77 (6). Anal. Calc. for $C_{24}H_{21}N$: C, 89.12; H, 6.54; N, 4.33. Found: C, 89.15; H, 6.51; N, 4.22.

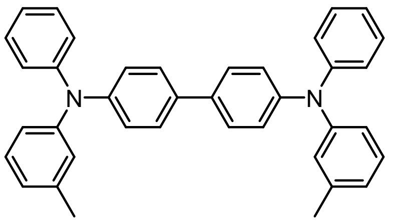

N^4,$N^{4'}$-diphenyl-N^4,$N^{4'}$-di-*m*-tolyl-[1,1'-biphenyl]-4,4'-diamine (TPD) (3m) (CAS No. 65181-78-4)[S5]: 51% isolated yield. Isolated as colorless crystals (Eluent: hexane/toluene = 60/40, R_f = 0.49). ^1H NMR (500 MHz, $CDCl_3$, TMS): δ 7.40–7.39 (m, 4H, Ar), 7.21–7.18 (m, 4H, Ar), 7.14–7.07 (m, 10H, Ar), 6.97–6.89 (m, 6H, Ar), 6.81–6.80 (m, 2H, Ar), 2.22 (s, 6H, CH_3). $^{13}C\{^1H\}$ NMR (125MHz, $CDCl_3$, TMS): δ 147.7, 147.6, 146.7, 139.0, 134.5, 129.1, 129.0, 127.2, 125.0, 124.1, 124.0, 123.8, 122.6, 121.6, 21.4. These NMR spectral data accord with those previously reported.[S5]

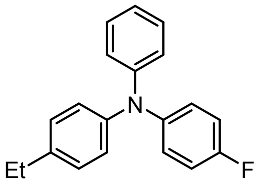

4-ethyl-*N*-(4-fluorophenyl)-*N*-phenylaniline (3n): 63% GC yield. Isolated as colorless crystals (Eluent: hexane/toluene = 19/1, R_f = 0.52). ^1H NMR (500 MHz, $CDCl_3$, TMS): δ 7.26–7.20 (m, 3H, Ar), 7.09–6.92 (m, 10H, Ar), 2.61 (q, J = 7.6 Hz, 2H, CH_2), 1.23 (t, J = 7.6 Hz, 3H, CH_3). $^{13}C\{^1H\}$ NMR (125MHz, $CDCl_3$, TMS): δ 158.7 (d, J = 242 Hz, Ar), 148.1, 145.4, 144.1, 138.9, 129.1, 128.7, 126.0 (d, J = 8.4 Hz, Ar), 124.2, 122.9,

122.0, 115.9 (d, J = 22.8 Hz, Ar), 28.2, 15.5. MS (70 eV, EI): m/z (%): 292 (17), 291 (78) [M^+], 277 (22), 276 (100), 185 (5), 77 (8), 51 (5). Anal. Calc. for $C_{18}H_{21}FN$: C, 82.45; H, 6.23; F, 6.52; N, 4.81. Found: C, 82.26; H, 6.45; N, 4.46.

Additional references

S1 X. Tao, L. Li, Y. Zhou, X. Qian, M. Zhao, L. Cai and X. Xie, *Chin. J. Chem.*, 2017, **35**, 1749.

S2 Y. Hirai and Y. Uozumi, *Chem. Commun.*, 2010, **46**, 1103.

S3 M. Kurt, S. Okur, S. Demic, J. Karpagam and N. Sunderganes, *J. Raman Spectrosc.*, 2011, **42**, 1682.

S4 A. Tsubouchi, D. Muramatsu and T. Takeda, *Angew. Chem. Int. Ed.*, 2013, **52**, 12719.

S5 D. S. Surry and S. L. Buchwald, *J. Am. Chem. Soc.*, 2007, **129**, 10354.

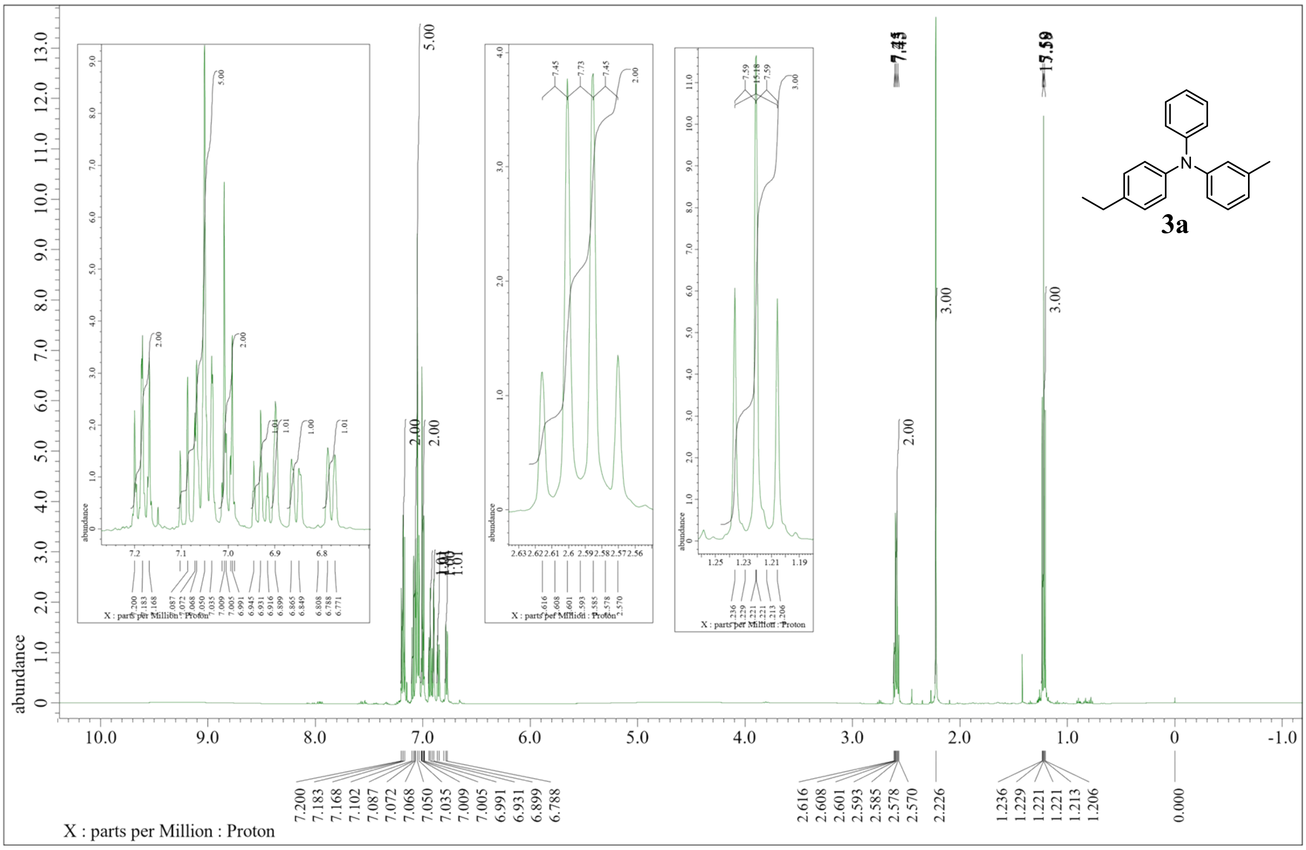

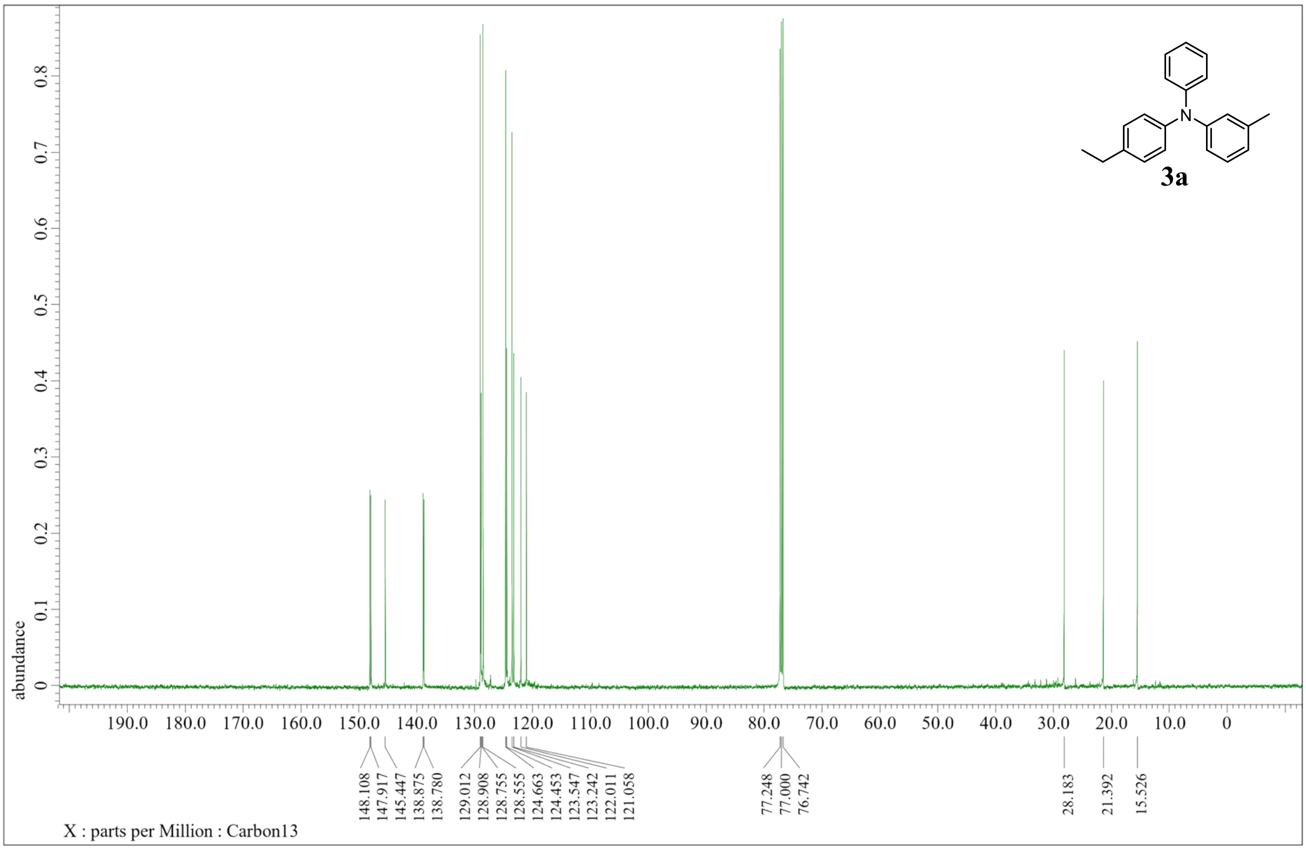

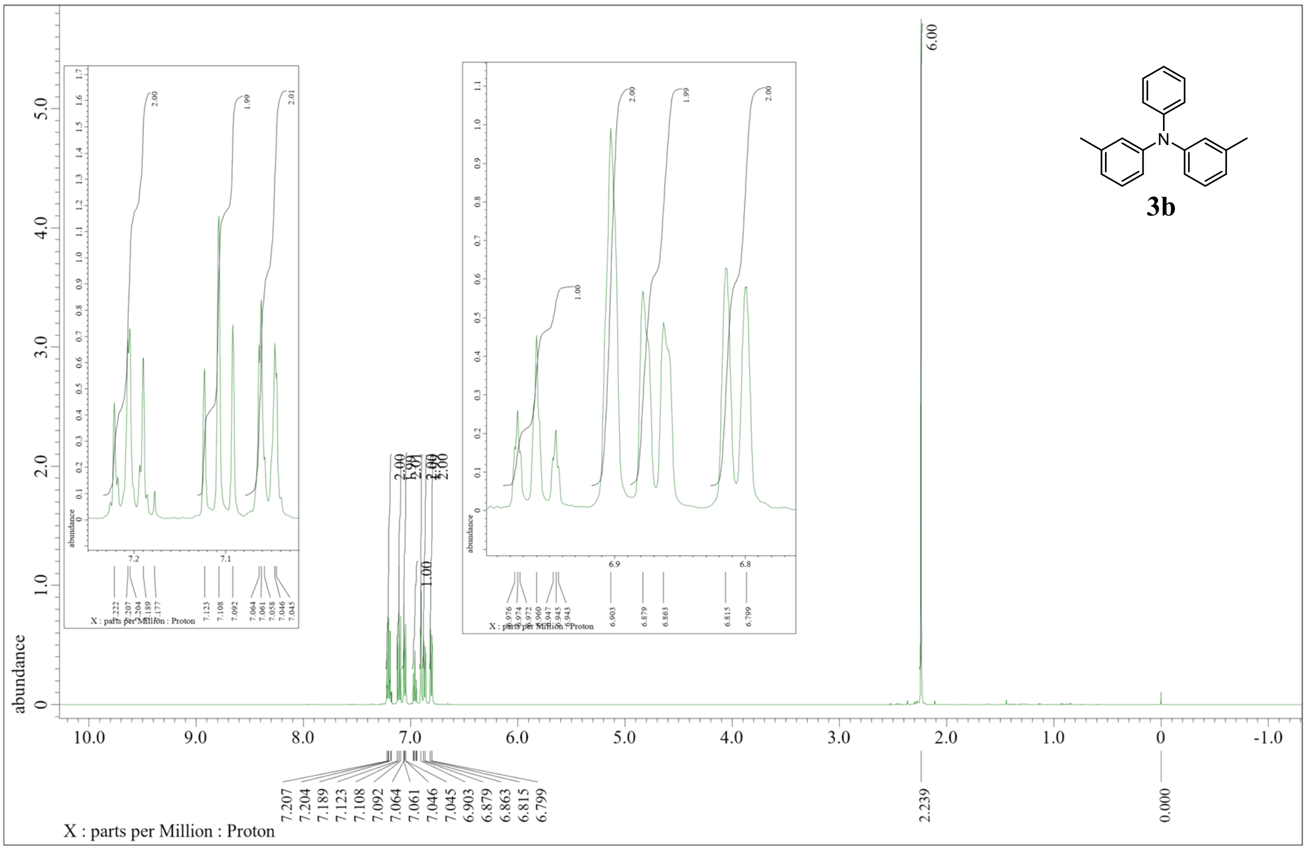

3b
abundance
X : parts per Million : Proton
7.207
7.204
7.189
7.123
7.108
7.092
7.064
7.061
7.046
7.045
6.903
6.879
6.863
6.815
6.799
2.239
0.000
6.00

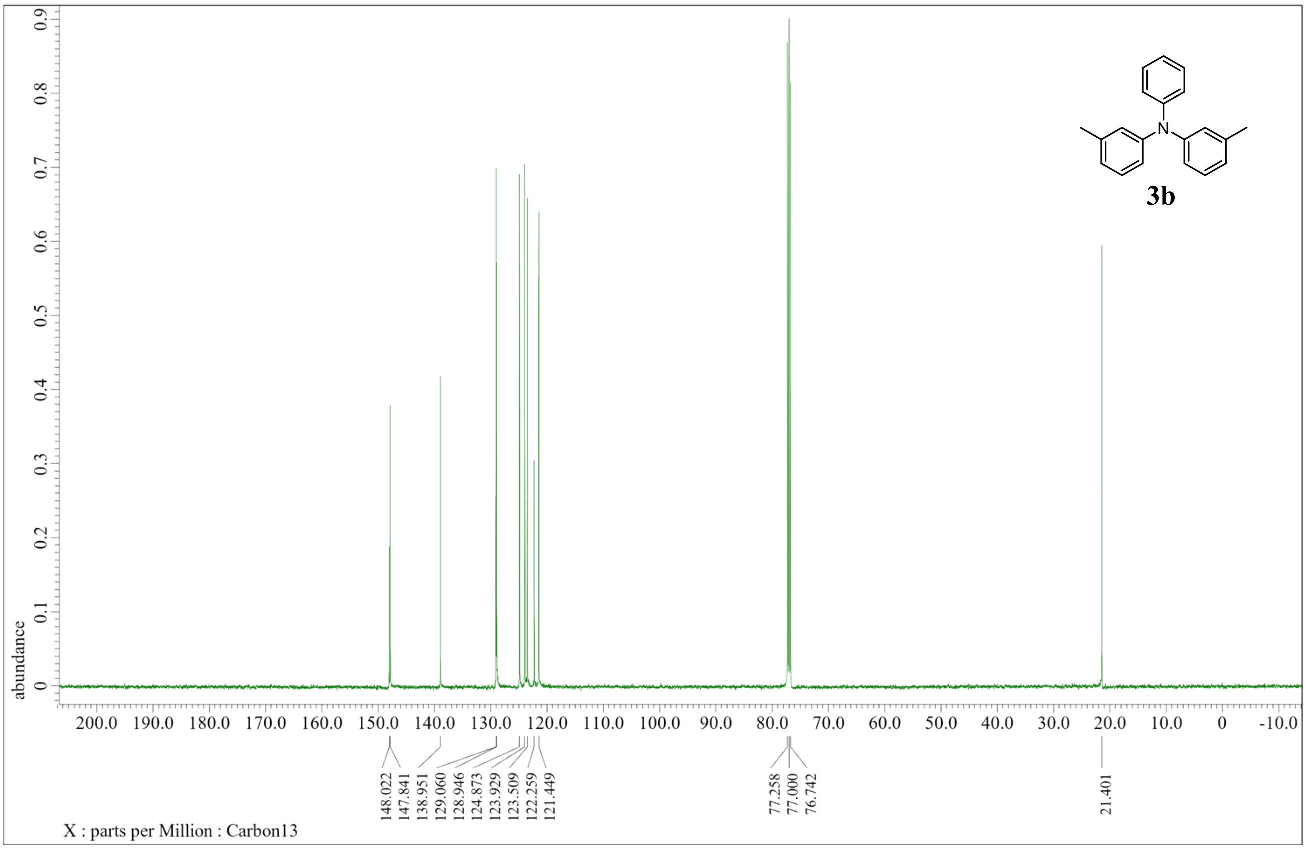

3b
abundance
X : parts per Million : Carbon13
148.022
147.841
138.951
129.060
128.946
124.873
123.929
123.509
122.259
121.449
77.258
77.000
76.742
21.401

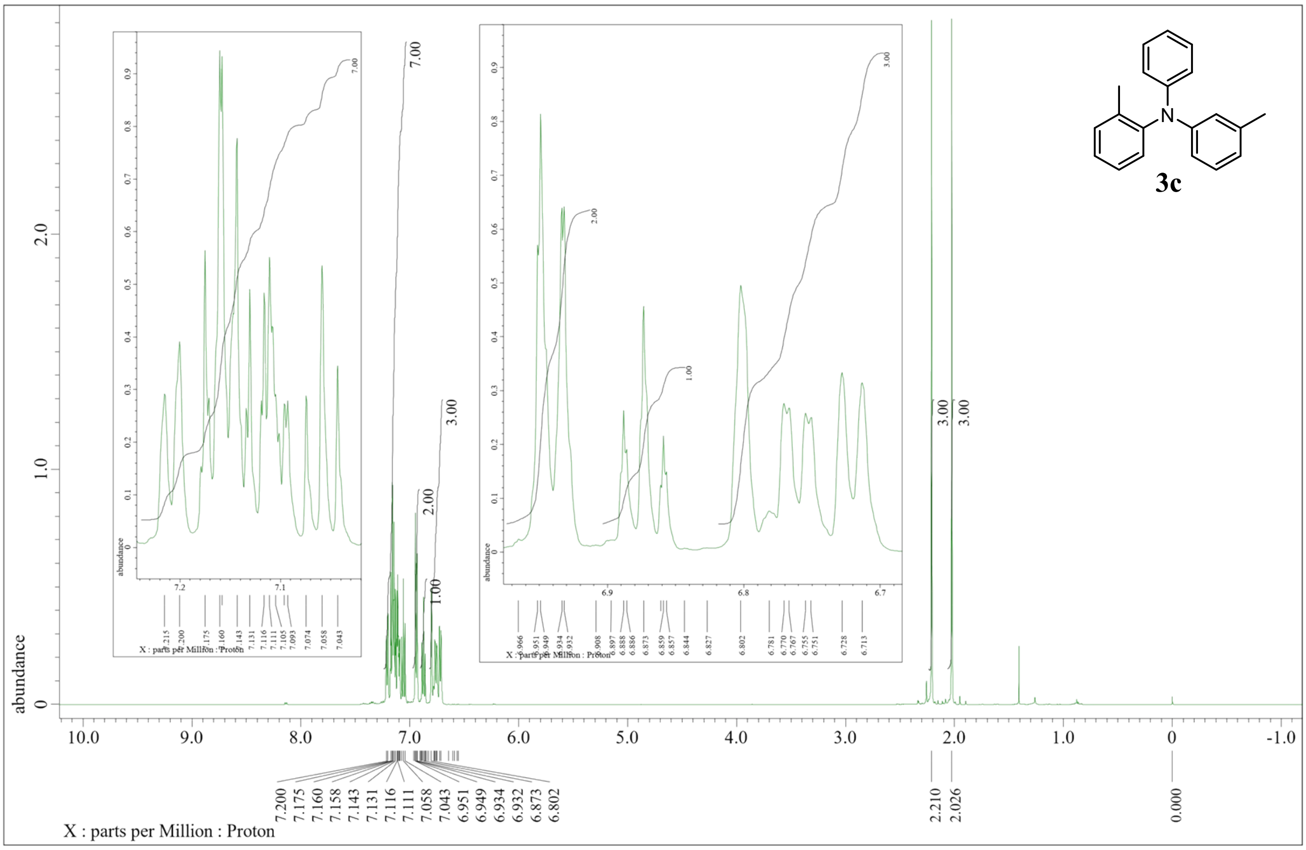

3c
abundance
X : parts per Million : Proton
7.200
7.175
7.160
7.158
7.143
7.131
7.116
7.111
7.058
7.043
6.951
6.949
6.934
6.932
6.873
6.802
2.210
2.026
0.000
7.00
2.00
1.00
3.00
3.00
3.00

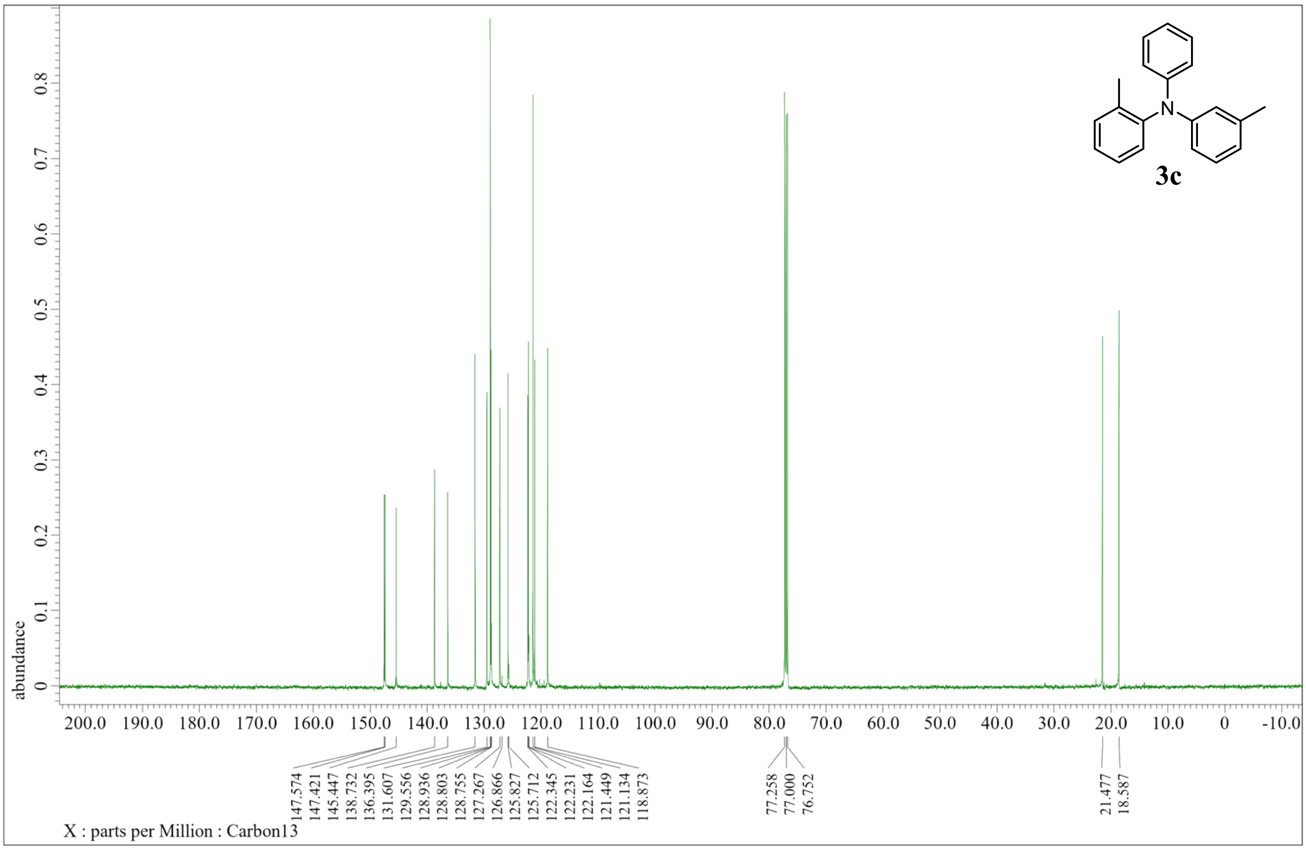

3c
abundance
X : parts per Million : Carbon13
147.574
147.421
145.447
138.732
136.395
131.607
129.556
128.936
128.803
128.755
127.267
126.866
125.827
125.712
122.345
122.231
122.164
121.449
121.134
118.873
77.258
77.000
76.752
21.477
18.587

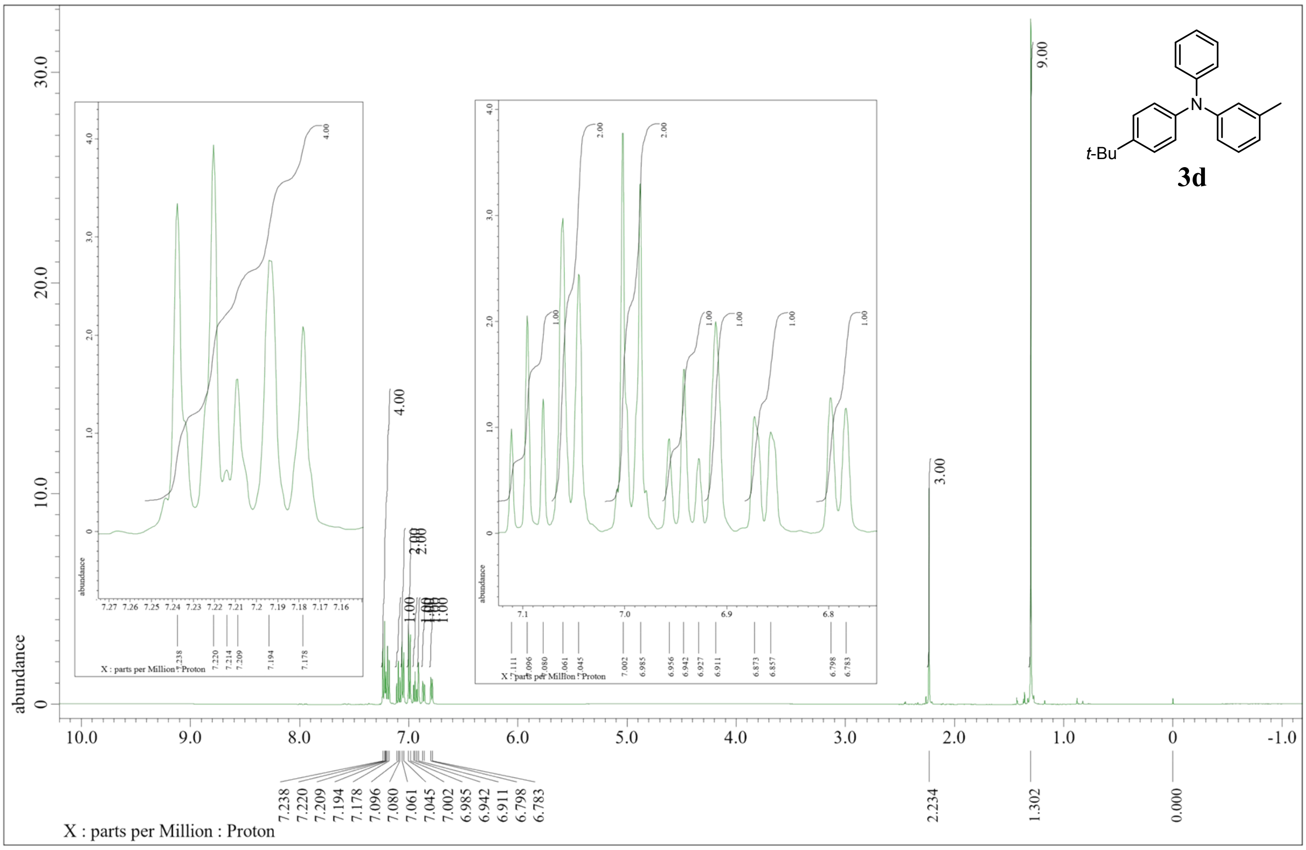
3d
t-Bu
abundance
X : parts per Million : Proton
7.238
7.220
7.209
7.194
7.178
7.096
7.080
7.061
7.045
7.002
6.985
6.942
6.911
6.798
6.783
2.234
1.302
0.000
4.00
3.00
9.00

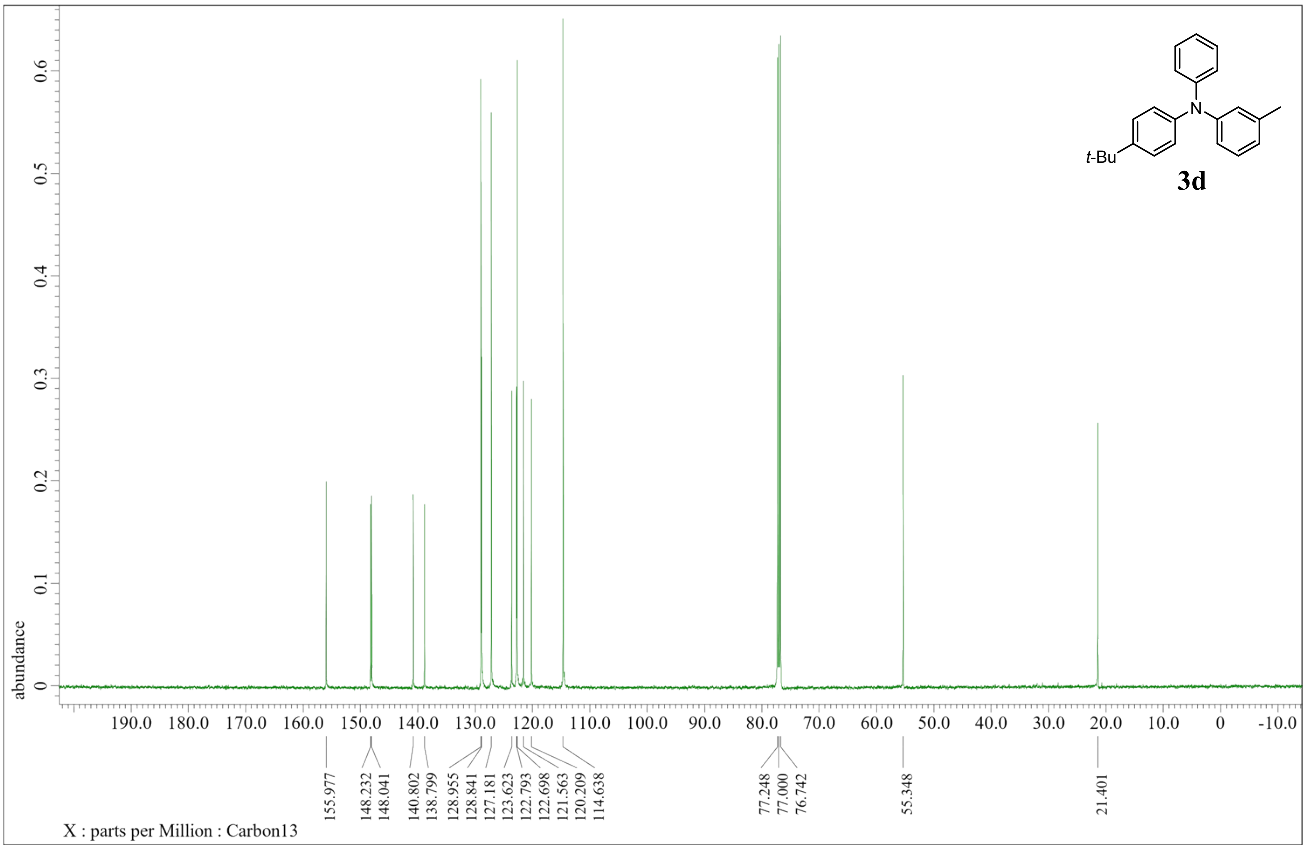
3d
t-Bu
abundance
X : parts per Million : Carbon13
155.977
148.232
148.041
140.802
138.799
128.955
128.841
127.181
123.623
122.793
122.698
121.563
120.209
114.638
77.248
77.000
76.742
55.348
21.401

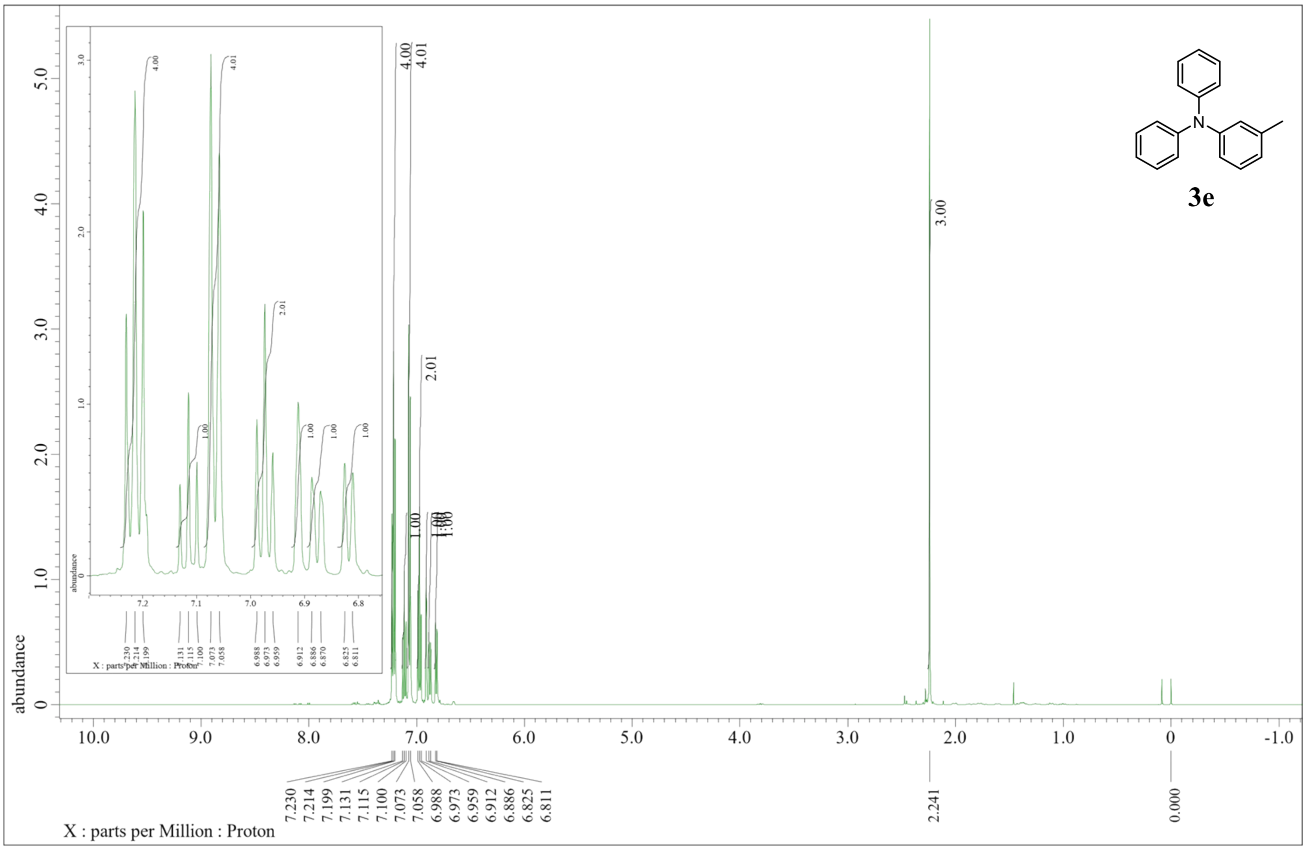
3e
abundance
X : parts per Million : Proton
4.00
4.01
2.01
1.00
1.00
1.00
1.00
3.00
7.230
7.214
7.199
7.131
7.115
7.100
7.073
7.058
6.988
6.973
6.959
6.912
6.886
6.825
6.811
2.241
0.000

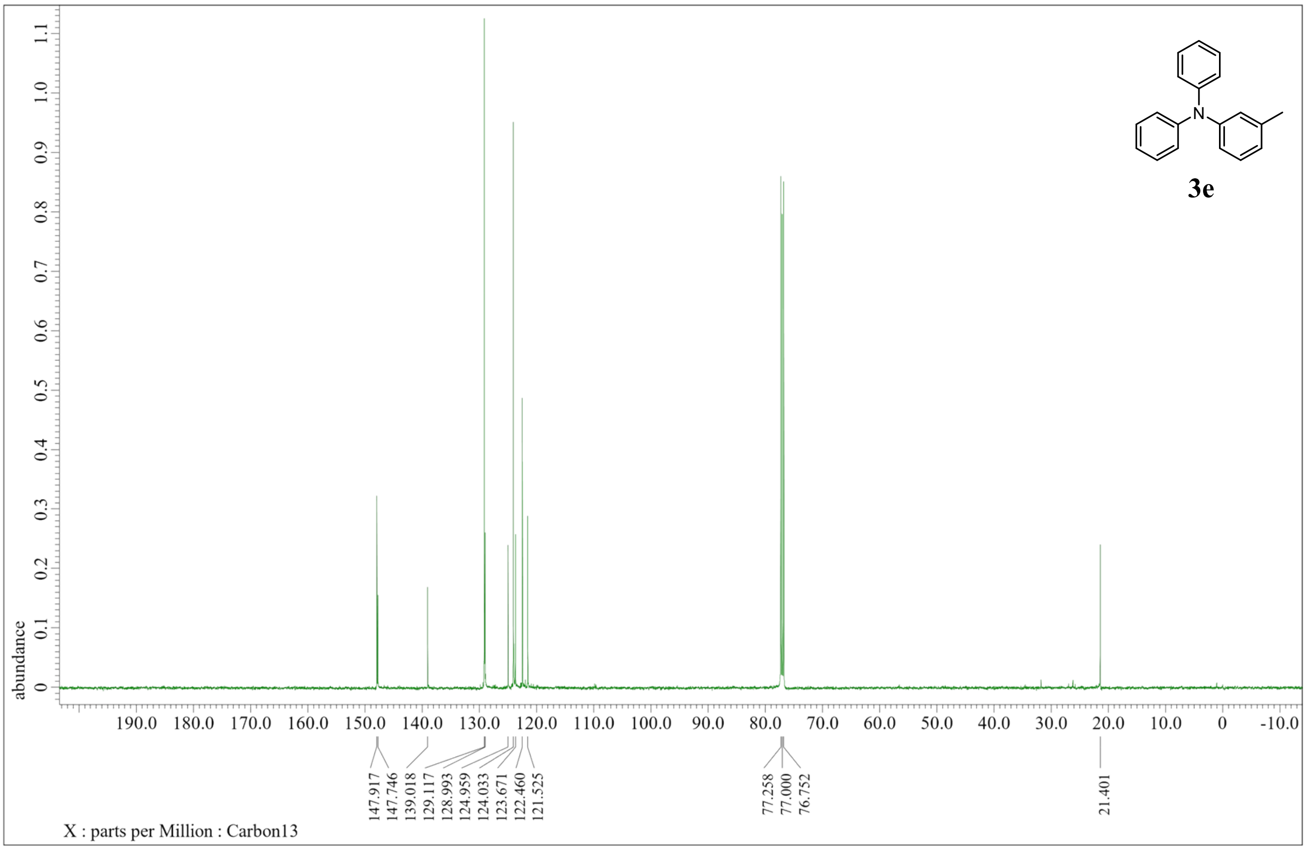
3e
abundance
X : parts per Million : Carbon13
147.917
147.746
139.018
129.117
128.993
124.959
124.033
123.671
122.460
121.525
77.258
77.000
76.752
21.401

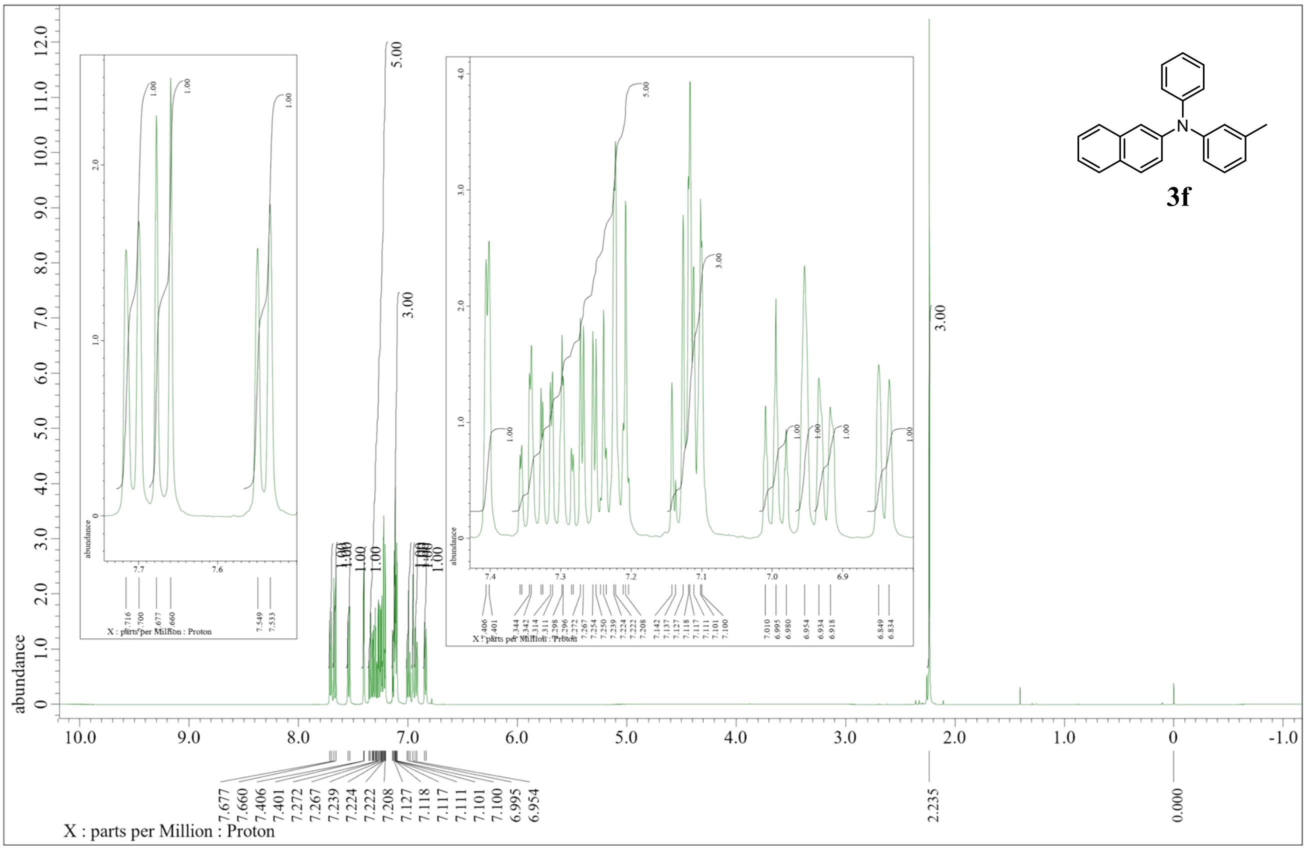

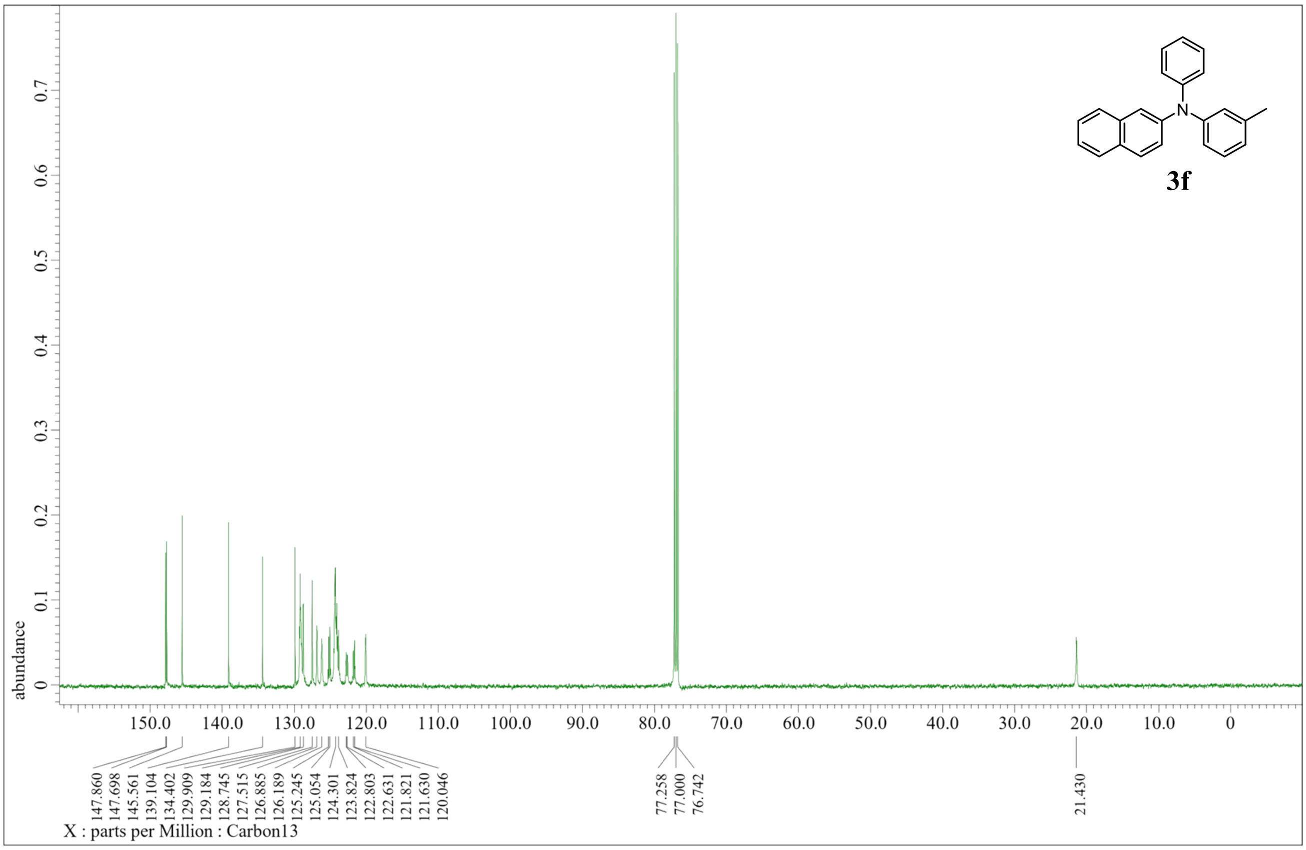

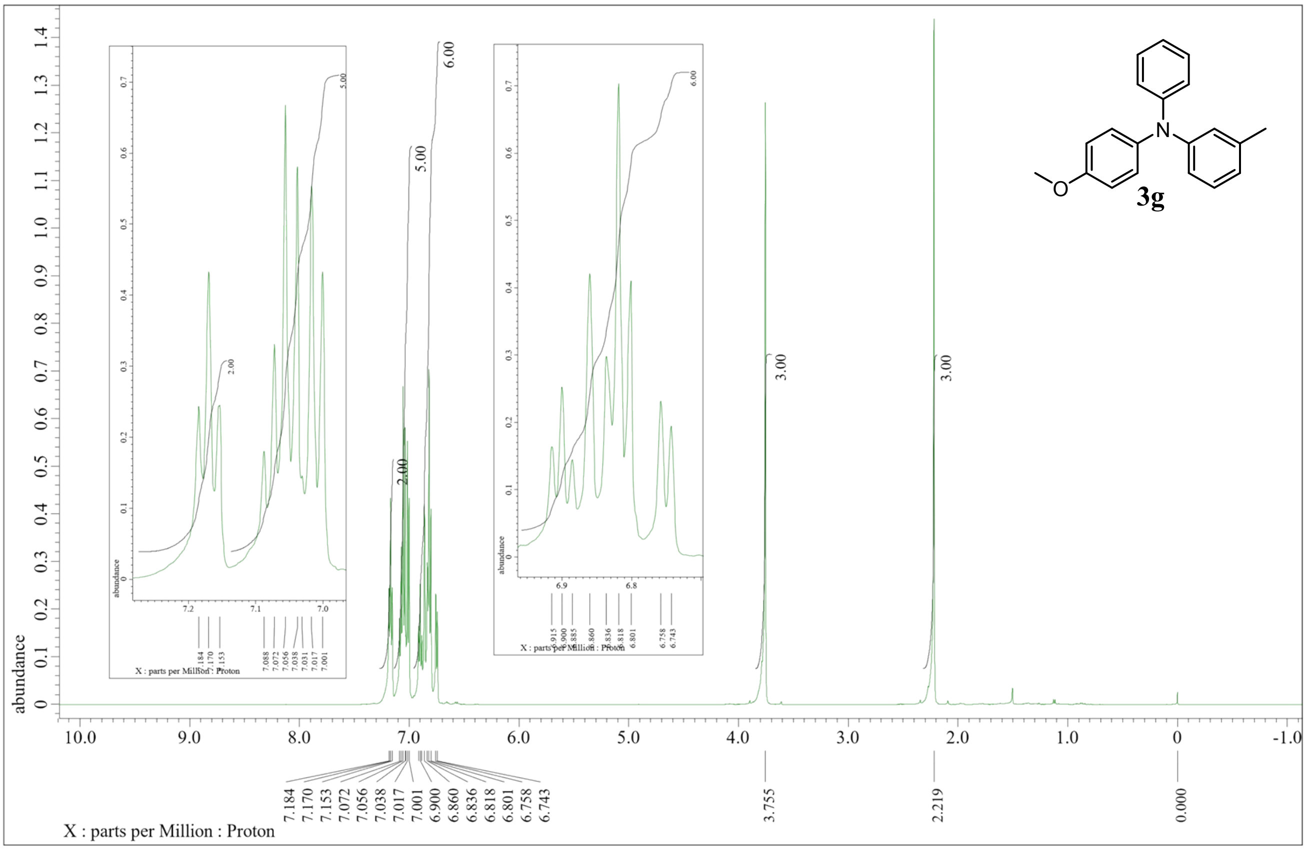

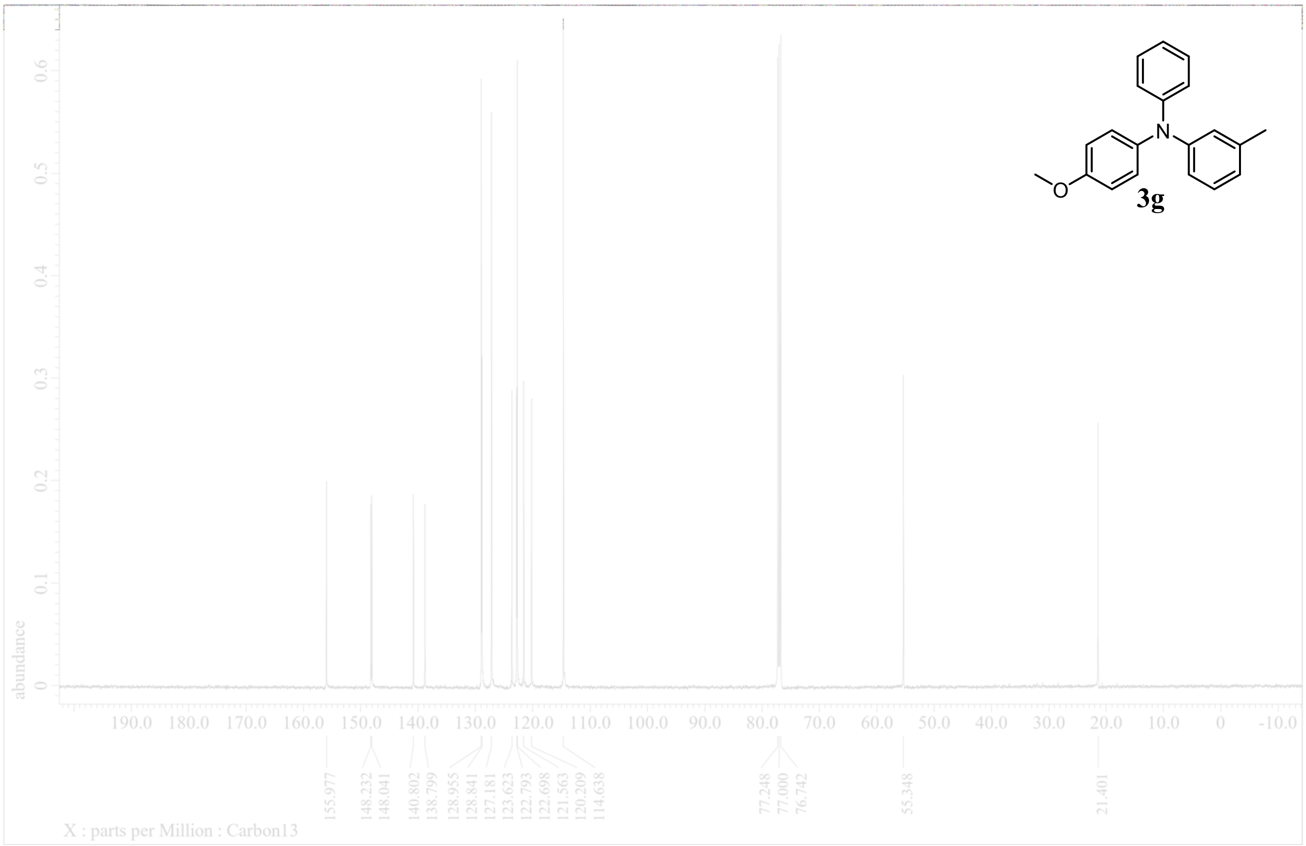

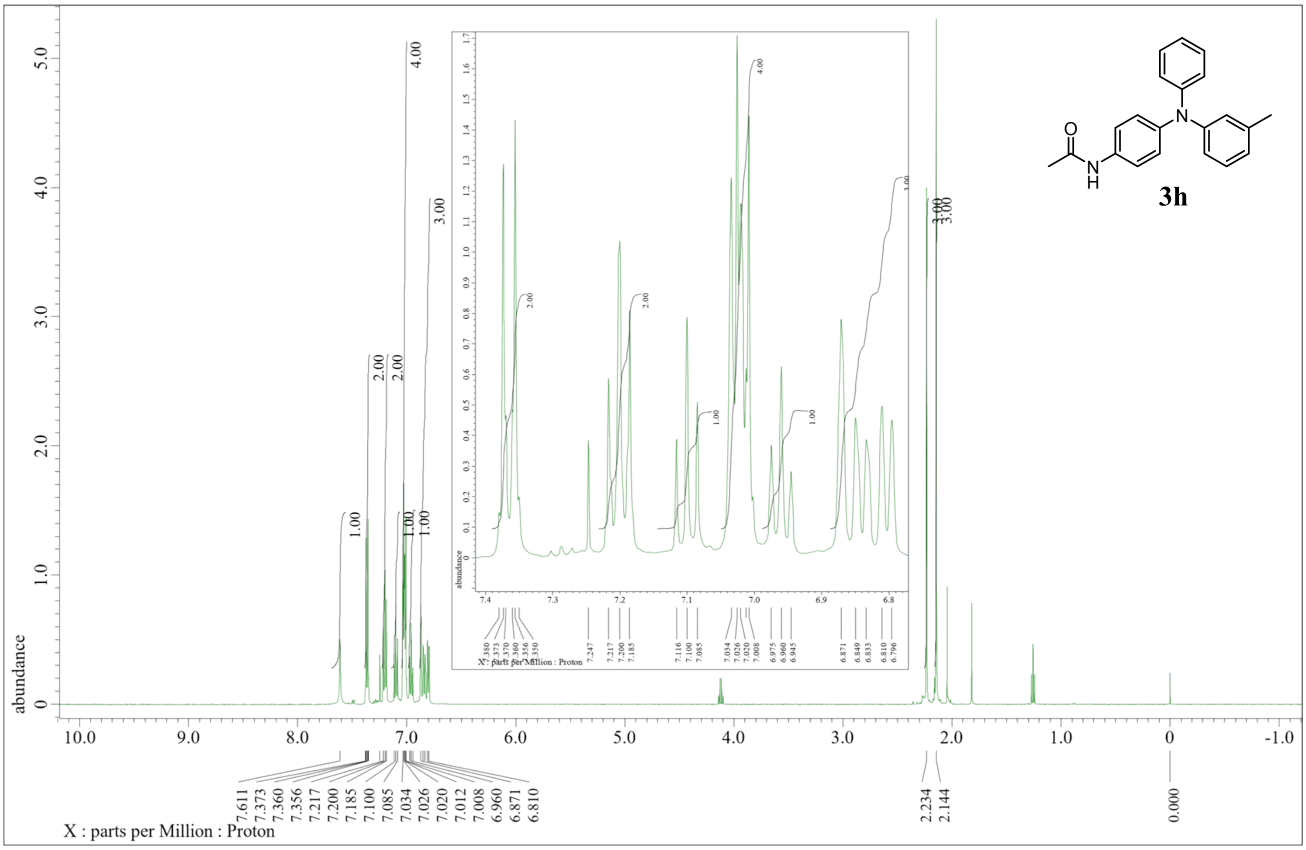
3h
abundance
X : parts per Million : Proton
7.611
7.373
7.360
7.356
7.217
7.200
7.185
7.100
7.085
7.034
7.026
7.020
7.012
7.008
6.960
6.871
6.810
2.234
2.144
0.000
1.00
2.00
2.00
1.00
1.00
4.00
3.00
3.00
3.00

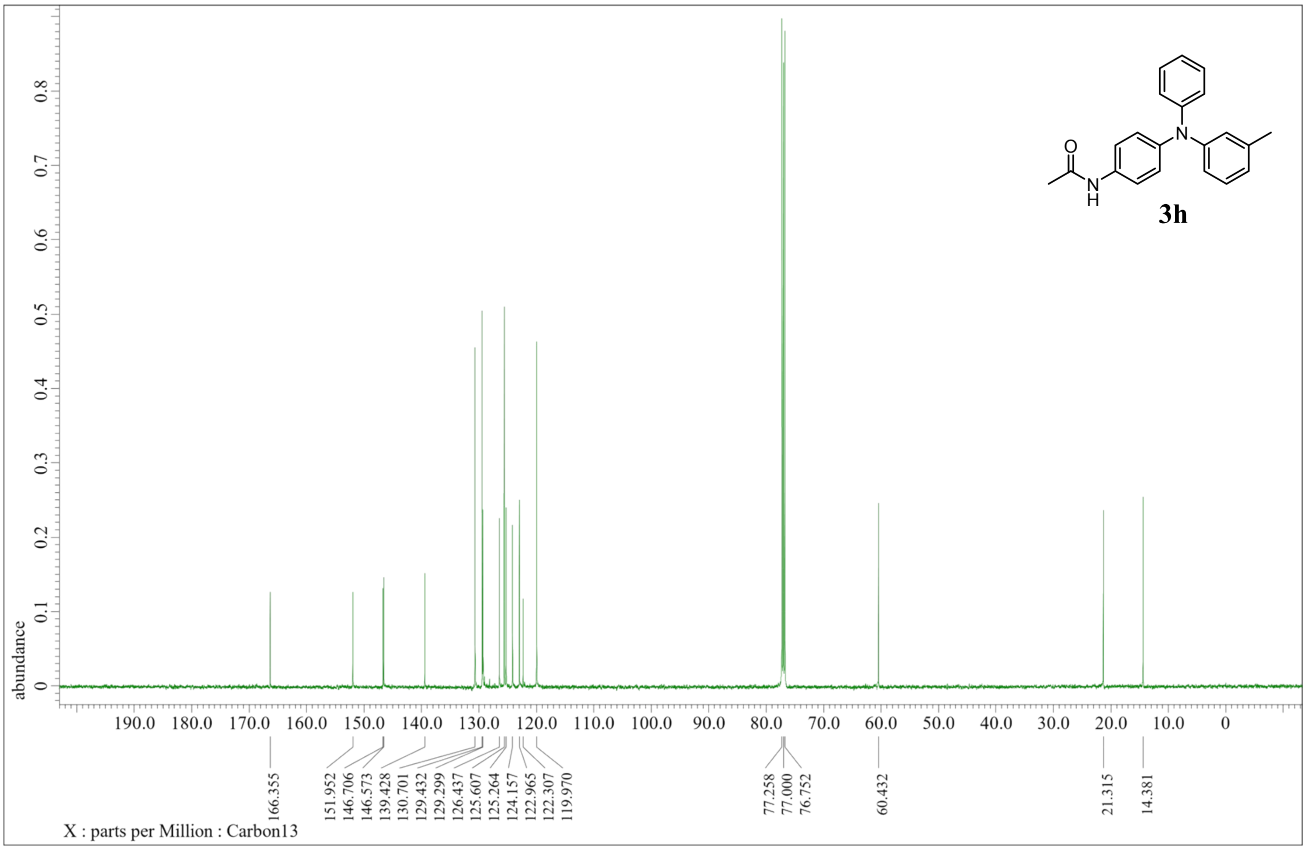
3h
abundance
X : parts per Million : Carbon13
166.355
151.952
146.706
146.573
139.428
130.701
129.432
129.299
126.437
125.607
125.264
124.157
122.965
122.307
119.970
77.258
77.000
76.752
60.432
21.315
14.381

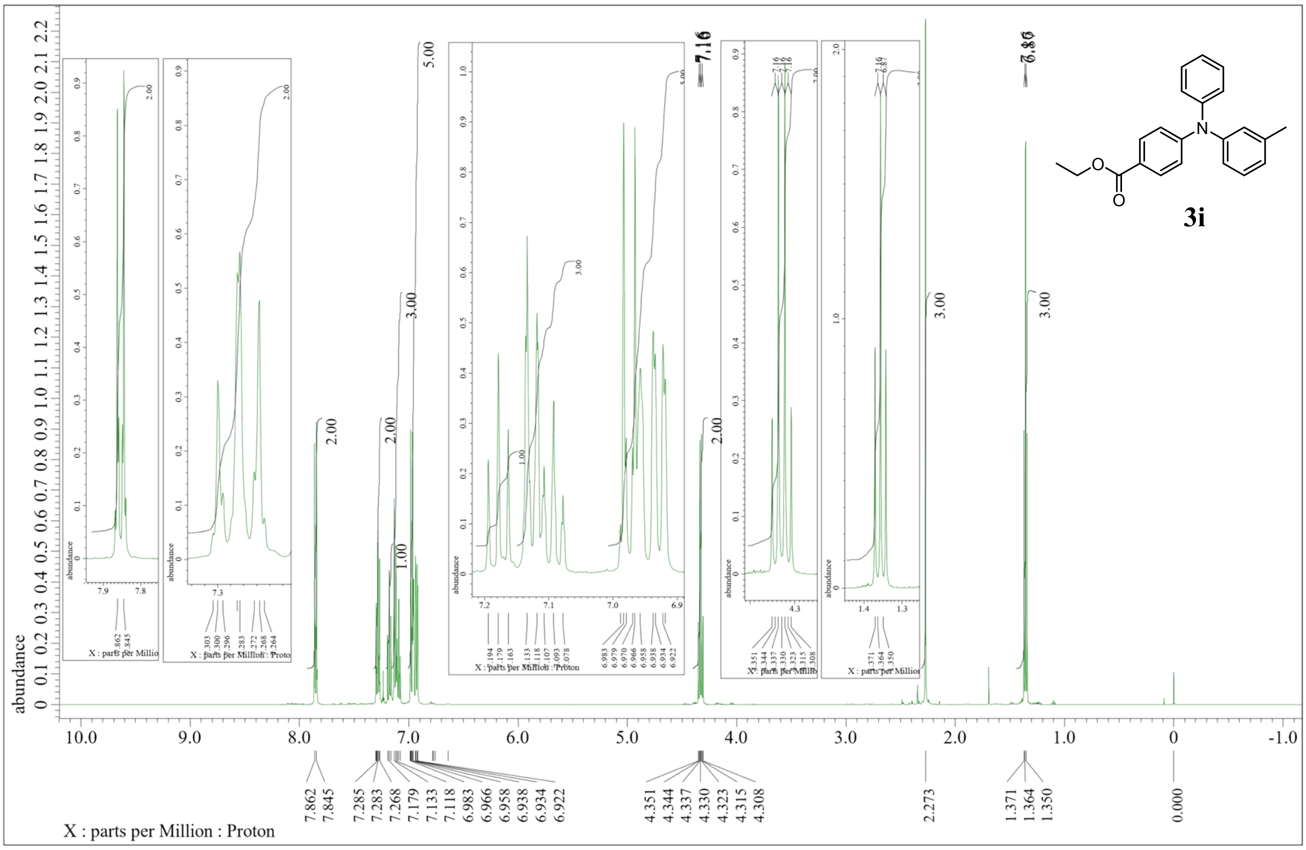
3i
X : parts per Million : Proton
abundance
7.862
7.845
7.285
7.283
7.268
7.179
7.133
7.118
6.983
6.966
6.958
6.938
6.934
6.922
4.351
4.344
4.337
4.330
4.323
4.315
4.308
2.273
1.371
1.364
1.350
0.000

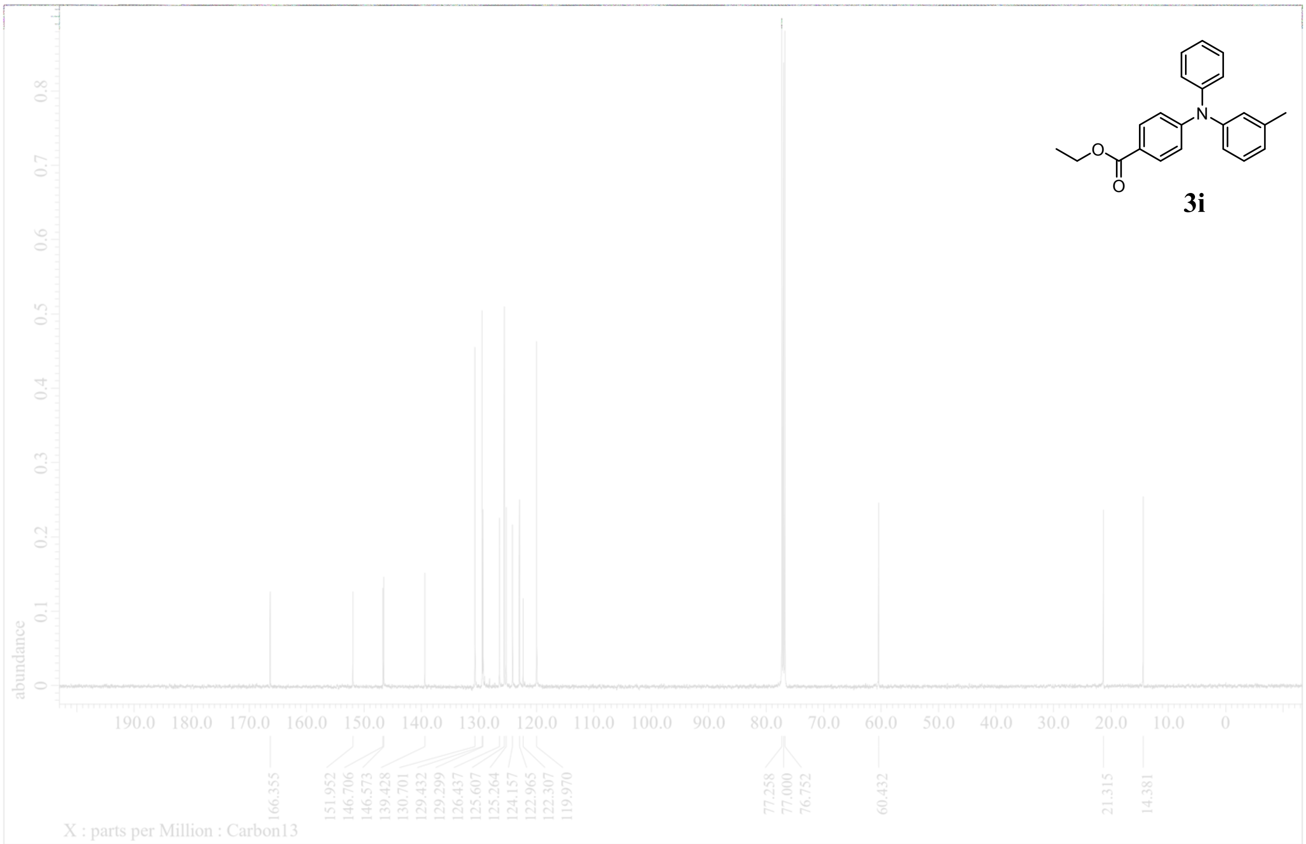
3i
X : parts per Million : Carbon13
abundance
166.355
151.952
146.706
146.573
139.428
130.701
129.432
129.299
126.437
125.607
125.264
124.157
122.965
122.307
119.970
77.258
77.000
76.752
60.432
21.315
14.381

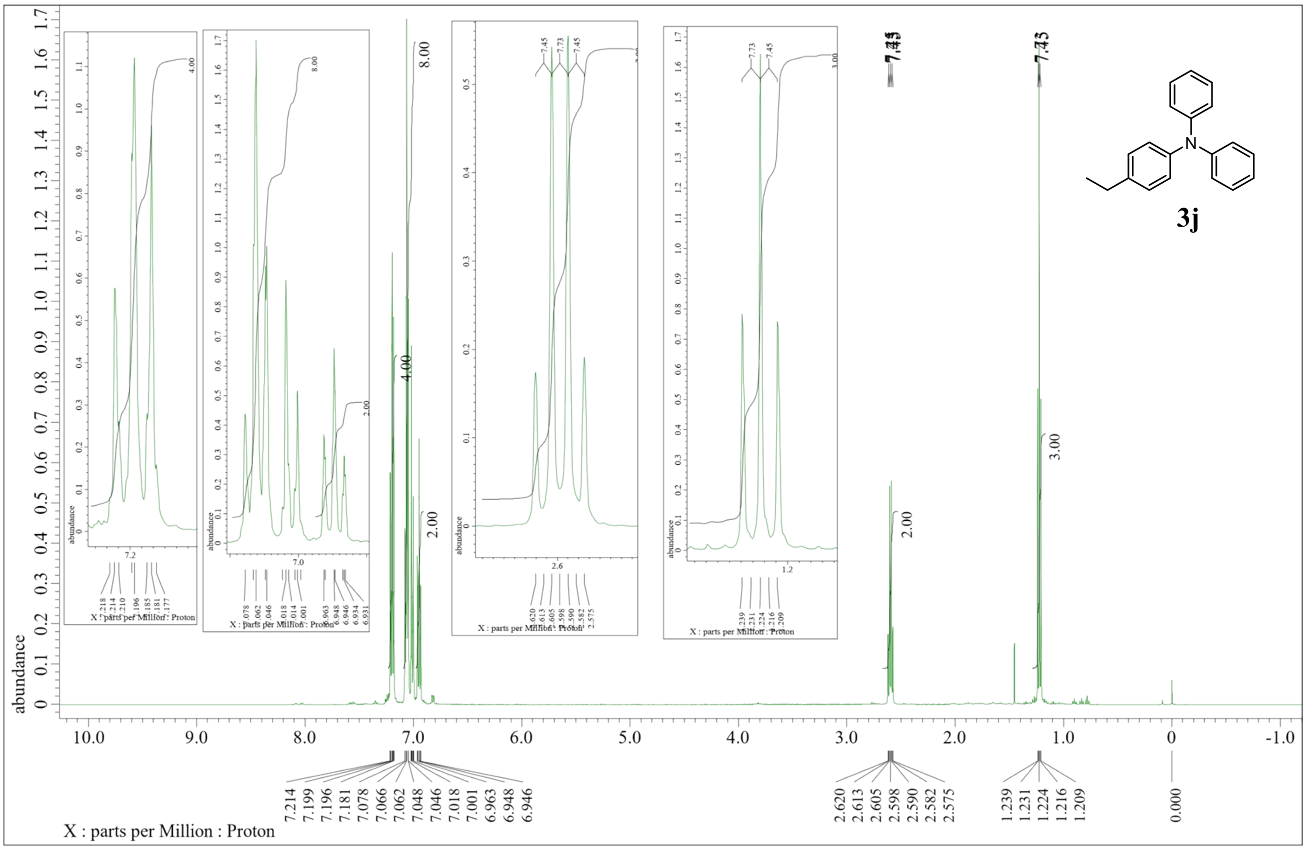
3j
X : parts per Million : Proton
abundance
8.00
4.00
2.00
2.00
3.00
7.214
7.199
7.196
7.181
7.078
7.066
7.062
7.048
7.046
7.018
7.001
6.963
6.948
6.946
2.620
2.613
2.605
2.598
2.590
2.582
2.575
1.239
1.231
1.224
1.216
1.209
0.000

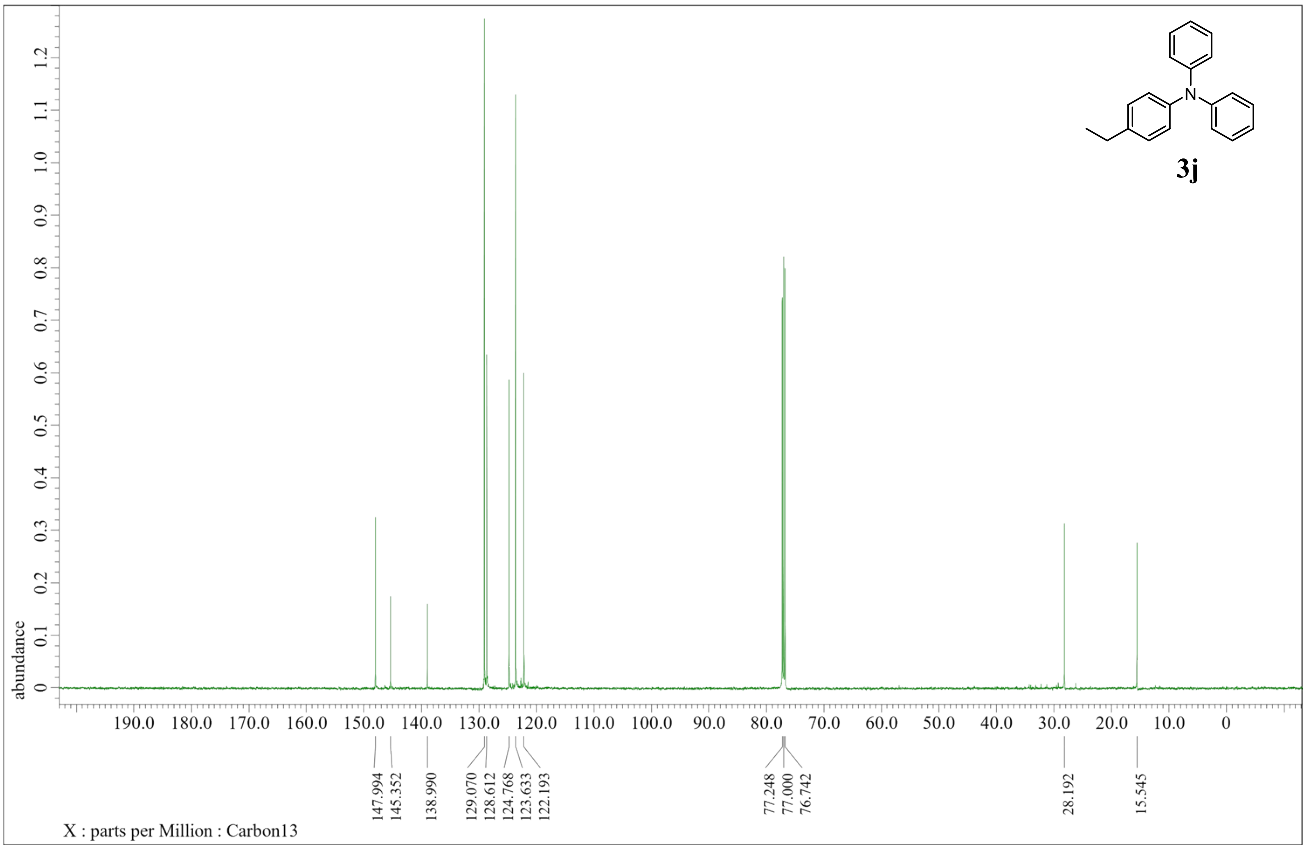
3j
X : parts per Million : Carbon13
abundance
147.994
145.352
138.990
129.070
128.612
124.768
123.633
122.193
77.248
77.000
76.742
28.192
15.545

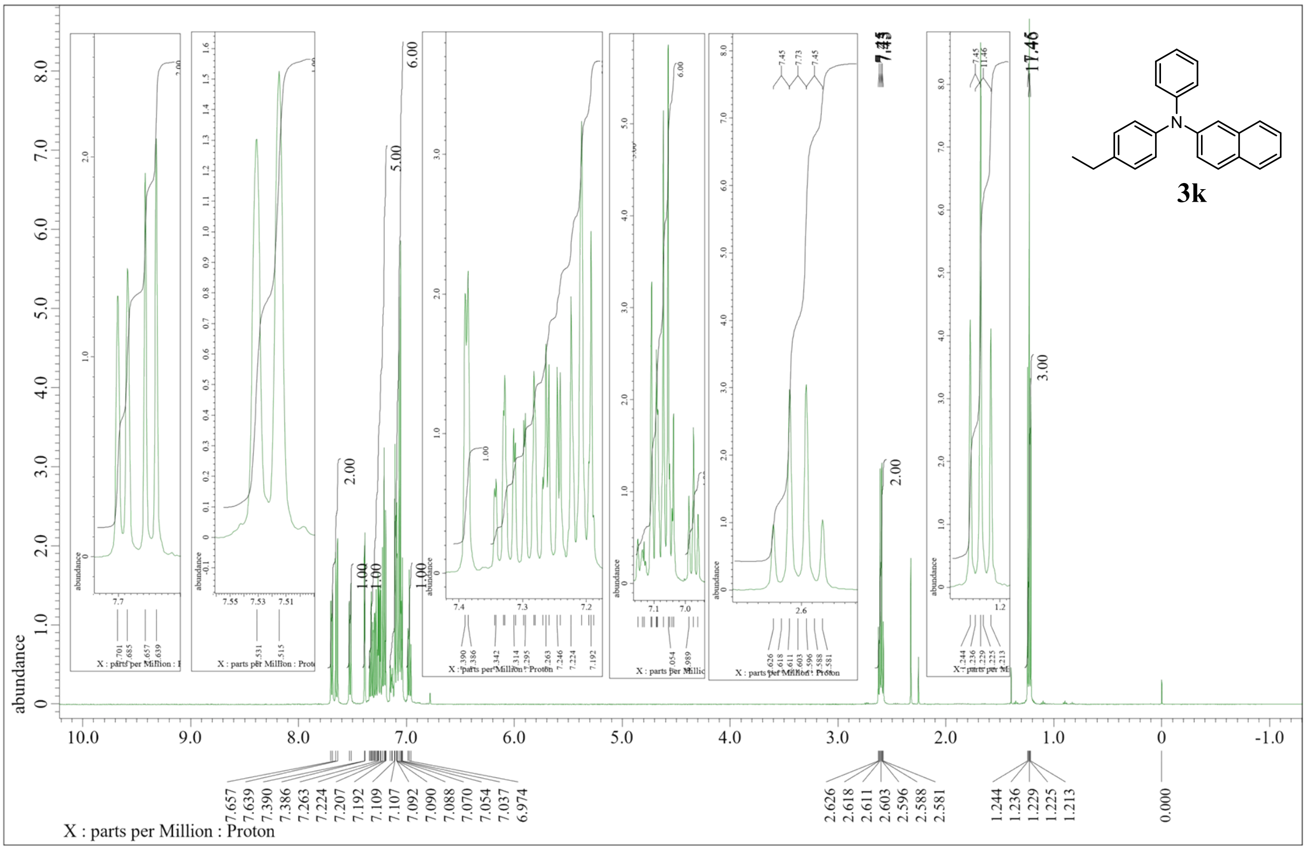
3k
X : parts per Million : Proton
abundance
7.657
7.639
7.390
7.386
7.263
7.224
7.207
7.192
7.109
7.107
7.092
7.090
7.088
7.070
7.054
7.037
6.974
2.626
2.618
2.611
2.603
2.596
2.588
2.581
1.244
1.236
1.229
1.225
1.213
0.000
2.00
1.00
1.00
5.00
6.00
1.00
2.00
3.00

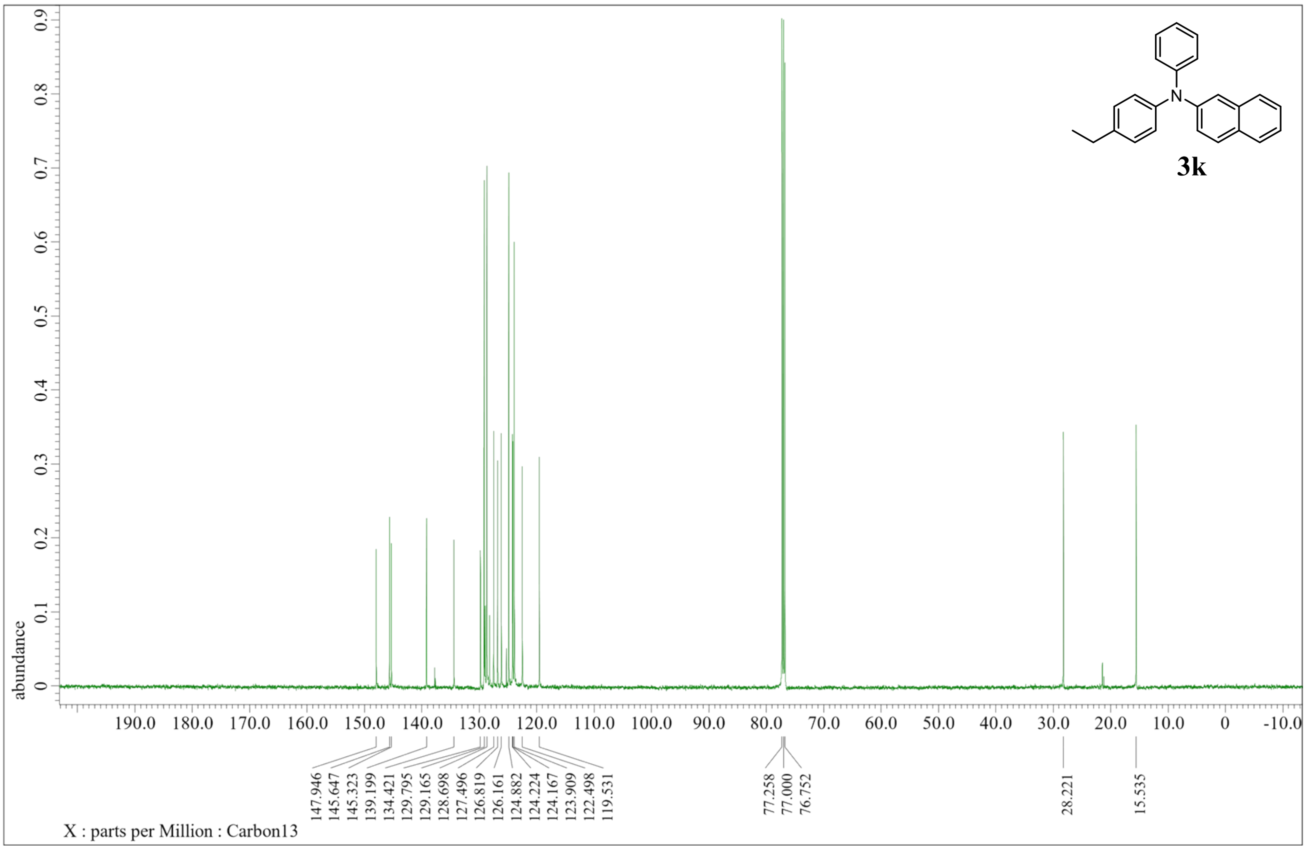
3k
X : parts per Million : Carbon13
abundance
147.946
145.647
145.323
139.199
134.421
129.795
129.165
128.698
127.496
126.819
126.161
124.882
124.224
124.167
123.909
122.498
119.531
77.258
77.000
76.752
28.221
15.535

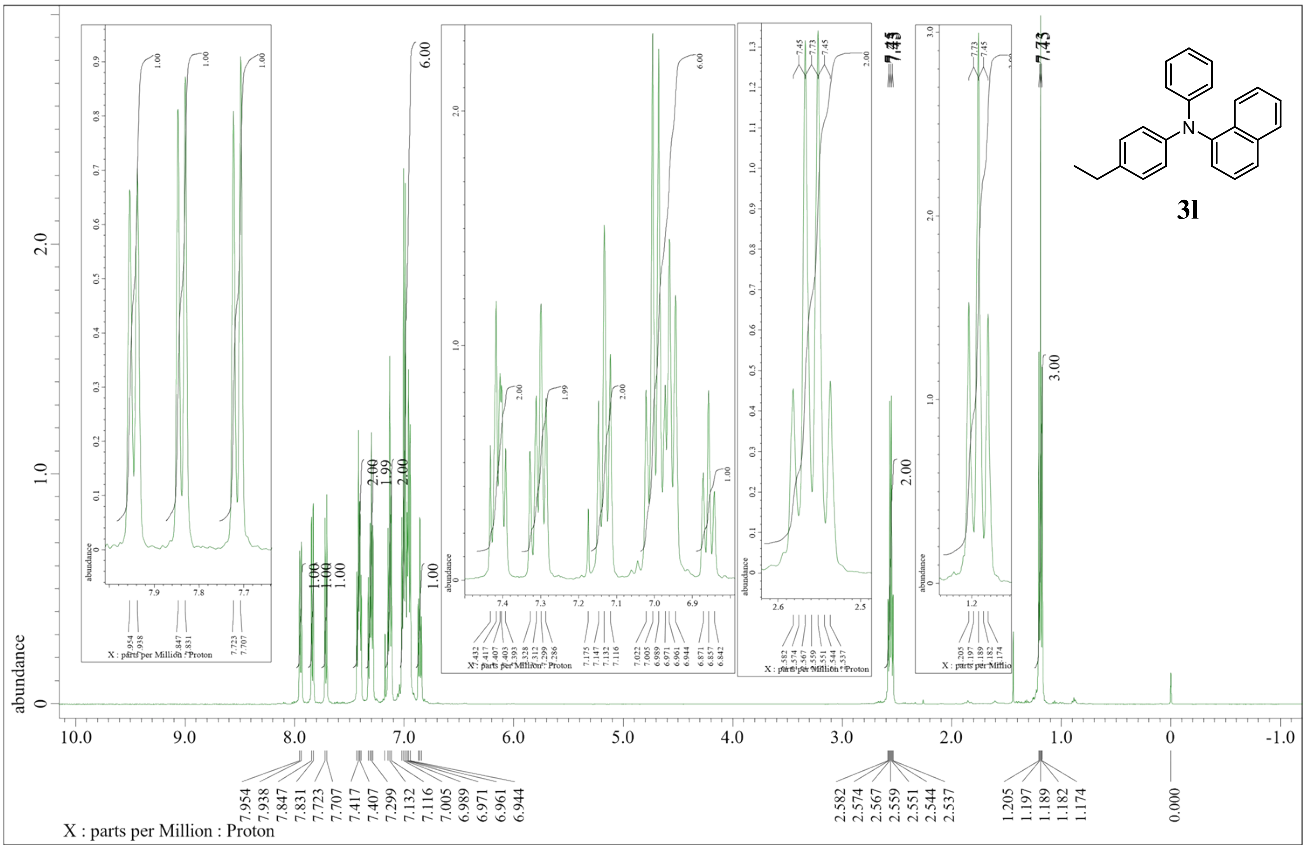
3l
X : parts per Million : Proton
abundance

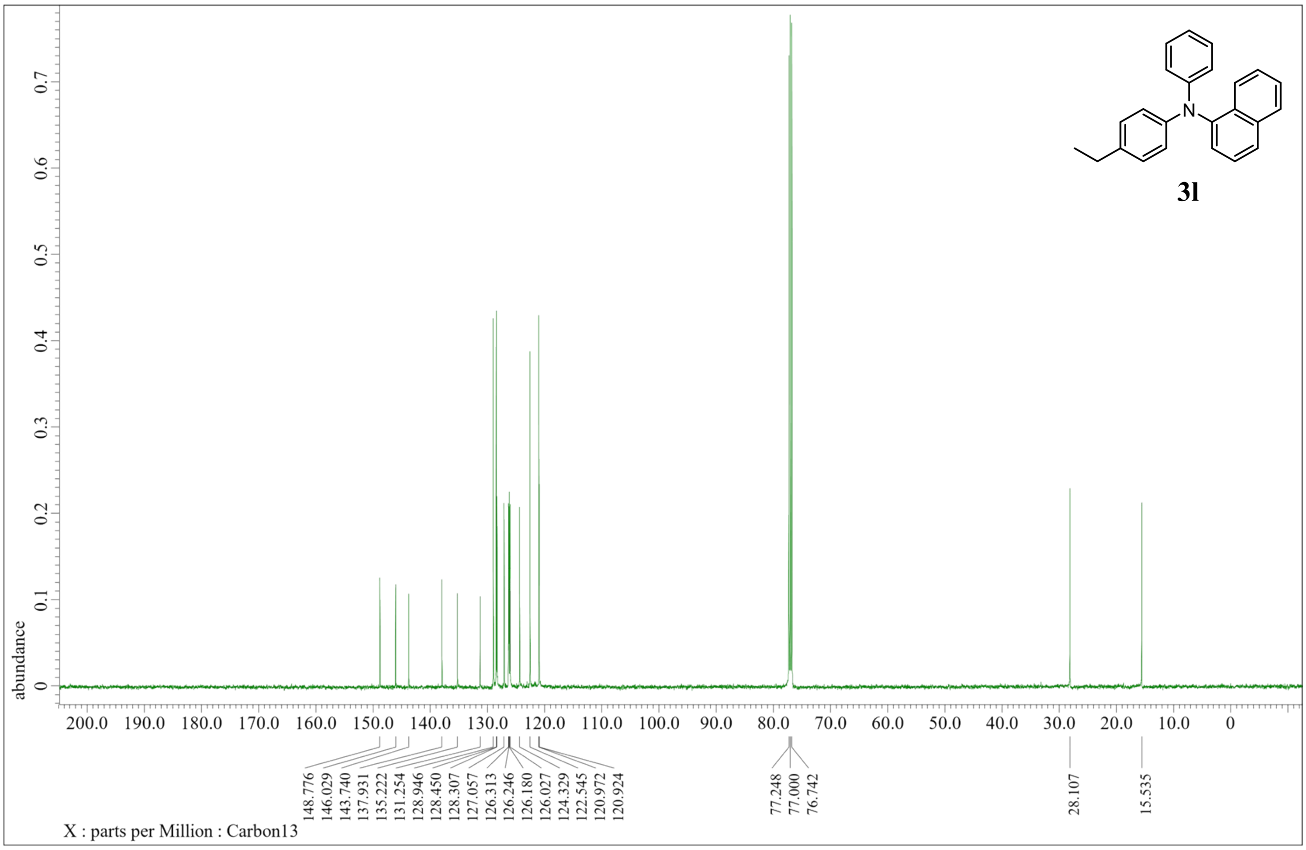
3l
X : parts per Million : Carbon13
abundance

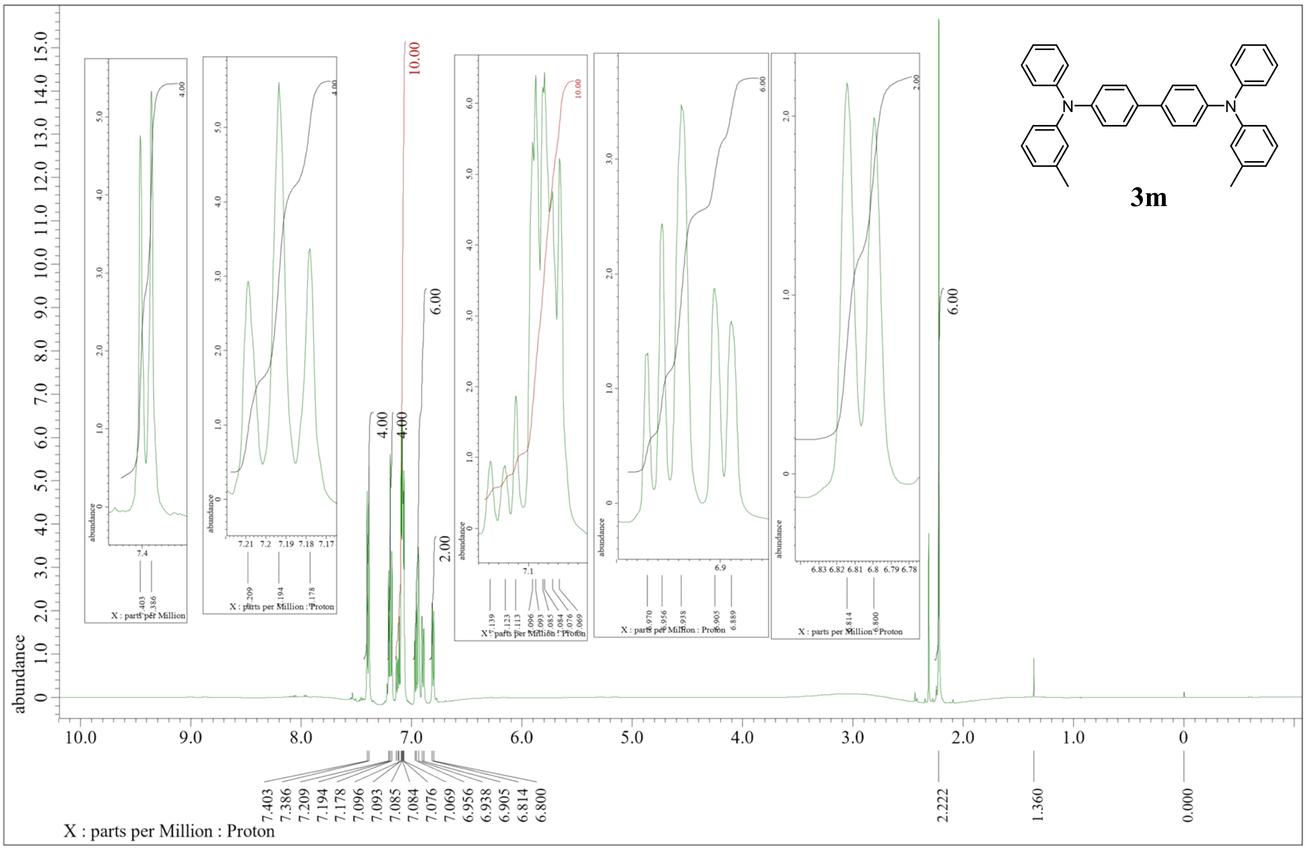
3m
abundance
X : parts per Million : Proton
7.403
7.386
7.209
7.194
7.178
7.096
7.093
7.085
7.084
7.076
7.069
6.956
6.938
6.905
6.814
6.800
2.222
1.360
0.000
4.00
4.00
10.00
6.00
2.00
6.00

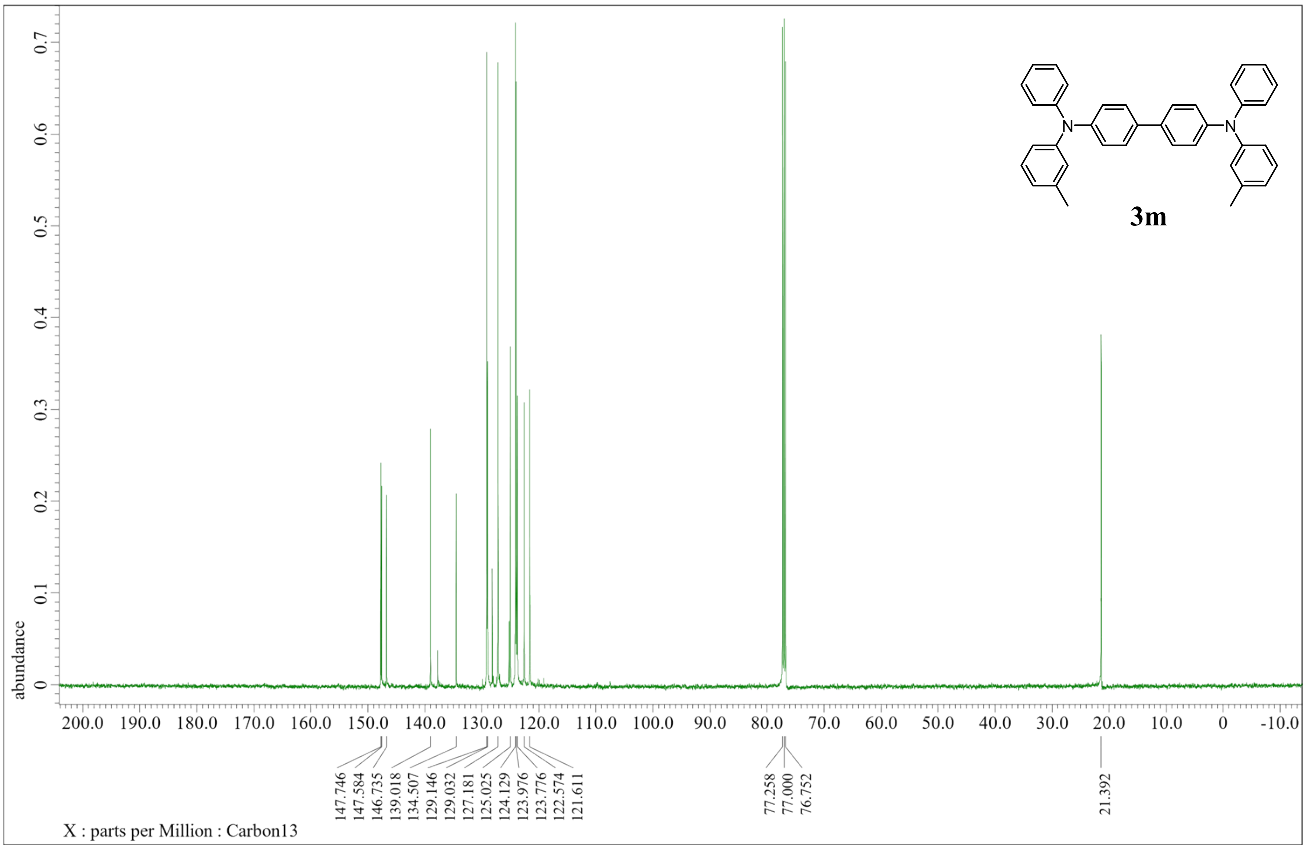
3m
abundance
X : parts per Million : Carbon13
147.746
147.584
146.735
139.018
134.507
129.146
129.032
127.181
125.025
124.129
123.976
123.776
122.574
121.611
77.258
77.000
76.752
21.392

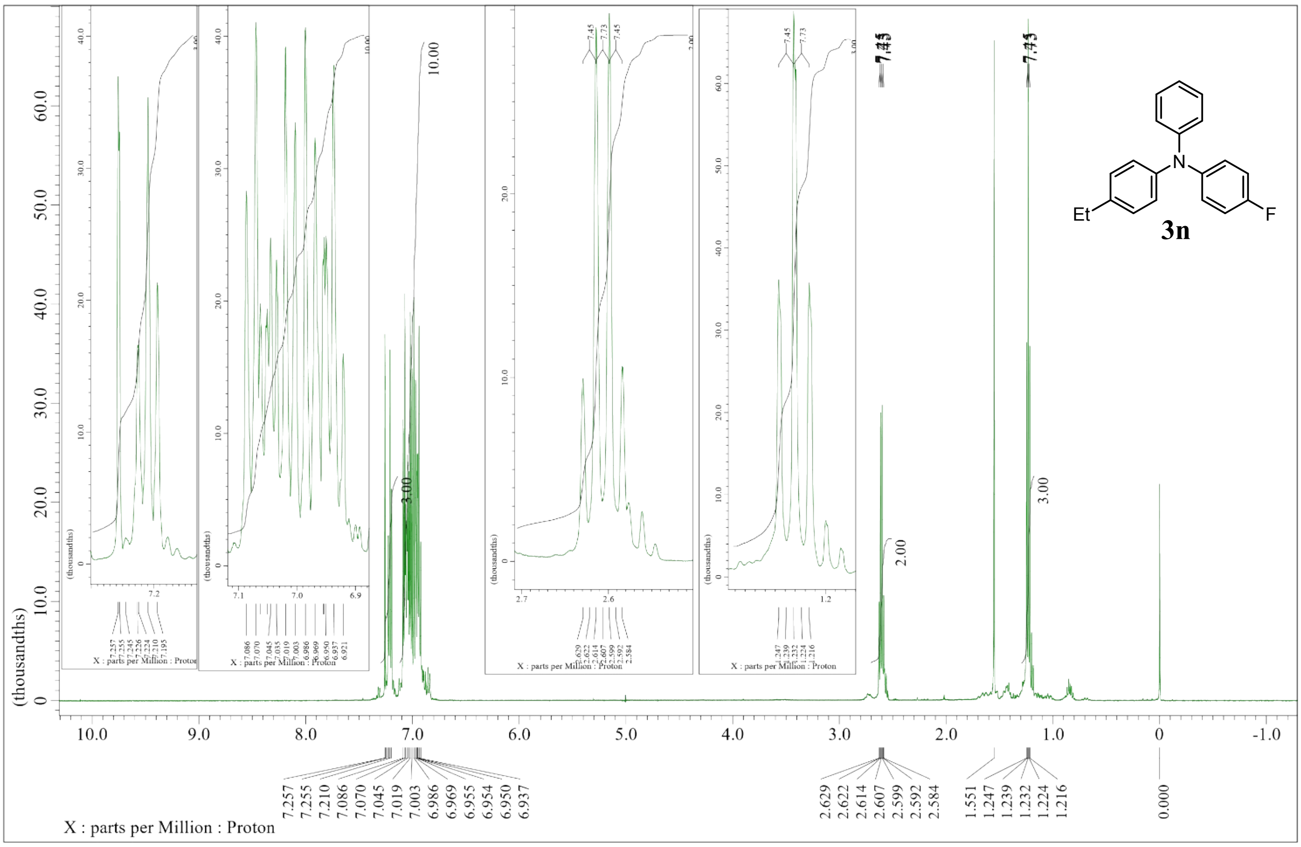

3n
Et
N
F
X : parts per Million : Proton
(thousandths)
7.257 7.255 7.210 7.086 7.070 7.045 7.019 7.003 6.986 6.969 6.955 6.954 6.950 6.937
2.629 2.622 2.614 2.607 2.599 2.592 2.584
1.551 1.247 1.239 1.232 1.224 1.216
0.000
10.00
3.00
2.00
3.00

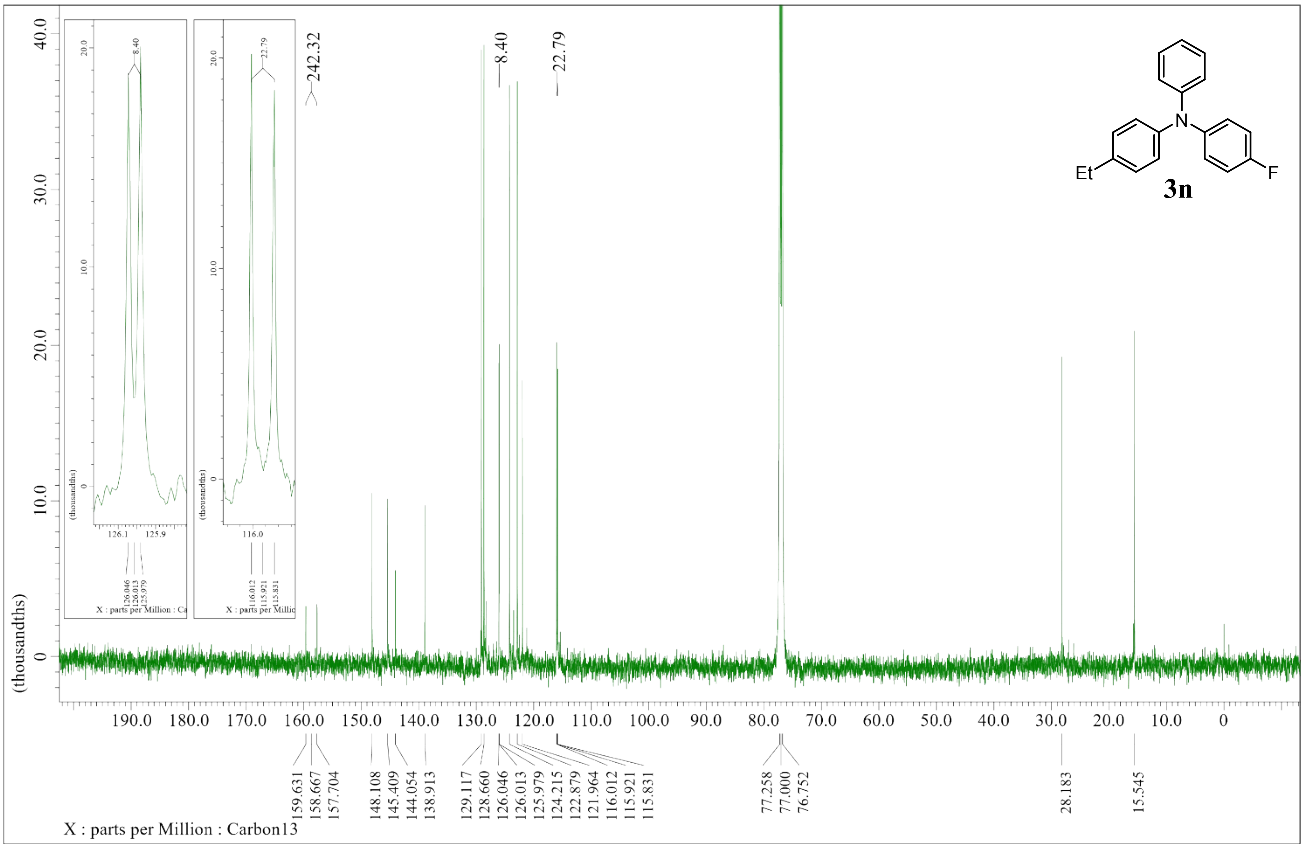

3n
Et
N
F
X : parts per Million : Carbon13
(thousandths)
242.32
8.40
22.79
159.631 158.667 157.704
148.108 145.409 144.054 138.913
129.117 128.660 126.046 126.013 125.979 124.215 122.879 121.964 116.012 115.921 115.831
77.258 77.000 76.752
28.183
15.545
